# Gut Microbiome Profiling in Eμ-TCL1 Mice Reveals Intestinal Changes and a Dysbiotic Signature Specific to Chronic Lymphocytic Leukemia

**DOI:** 10.1158/2767-9764.CRC-25-0022

**Published:** 2025-08-15

**Authors:** Sydney A. Skupa, Kathryn M. Cooper, Audrey L. Smith, Erin M. Drengler, Alexandria P. Eiken, Elizabeth Schmitz, Grace M. Waldron, Grinu Mathew, Mark Primeaux, Punita Dhawan, Geoffrey A. Talmon, Christopher R. D’Angelo, Dalia El-Gamal

**Affiliations:** 1 Eppley Institute for Research in Cancer and Allied Diseases, University of Nebraska Medical Center, Omaha, Nebraska.; 2 School of Interdisciplinary Informatics, University of Nebraska at Omaha, Omaha, Nebraska.; 3 Fred and Pamela Buffett Cancer Center, University of Nebraska Medical Center, Omaha, Nebraska.; 4 Department of Biochemistry and Molecular Biology, University of Nebraska Medical Center, Omaha, Nebraska.; 5 VA Nebraska-Western Iowa Health Care System, Omaha, Nebraska.; 6 Department of Pathology and Microbiology, University of Nebraska Medical Center, Omaha, Nebraska.; 7 Division of Hematology and Oncology, University of Nebraska Medical Center, Omaha, Nebraska.; 8 Division of Hematology and Oncology, Department of Internal Medicine, College of Medicine, University of Cincinnati, Cincinnati, Ohio.

## Abstract

The gut microbiome’s role in the pathogenesis of hematologic malignancies is actively being explored; yet studies on chronic lymphocytic leukemia (CLL) are limited. Using the Eμ-TCL1 murine model of CLL, we identify a unique and dysbiotic disease-associated gut microbiome that develops in mice over time. Leukemic mice show an increase in abundance of pathogenic bacteria, specifically members of the *Proteobacteria* phylum. We found that this dysbiotic microenvironment is associated with CLL involvement within the intestinal tract and high levels of markers indicative of altered tight junction permeability (e.g., claudin-2, soluble CD14, and zonulin). Moreover, utilizing the syngeneic model of CLL in tandem with an antibiotic-mediated microflora ablation approach, we found that leukemic mice receiving microflora-ablating antibiotics show marked changes to the gut microbiome and a delayed disease onset compared with mice receiving antibiotic-free water. Immunophenotyping of murine spleens showed that this delay in disease was accompanied by more tumor-reactive CD8^+^ T cells that coexpressed fewer inhibitory receptors (e.g., PD-1, LAG-3, and TIM-3). These findings confirm that a dysbiotic gut microbiome develops during CLL disease and demonstrate unique intestinal involvement and potential immune dysregulation occurring during CLL progression that may be influencing the overall microbial signature.

**Significance:**

There is a growing appreciation for the gut microbiome’s role in hematologic malignancies. Despite this, its role in CLL remains obscure. This study demonstrates a dysbiotic microbiome within CLL that may contribute to further intestinal and immune dysregulation present during CLL progression.

## Introduction

The gut microbiome is home to a community of microorganisms such as bacteria, fungi, and viruses (1–3). The gut microbiome has been well-recognized as an enabling characteristic of cancer with direct interactions between established cancer hallmarks, including tumor inflammation, immune evasion, genome instability, and resistance to therapies. Factors affecting the composition of the gut microbiome (e.g., diet, antibiotics, and living conditions) consistently affect the microbiome’s role in carcinogenesis, partially through inducing a phenomenon known as dysbiosis (3, 4). Gut dysbiosis, or the pathologic disruption of a stable microbiome community’s membership and function, can cause a plethora of complications such as disruption of epithelial tight junction barriers, increase of bacterial translocation–promoting inflammation and local immunosuppression, and alterations in microbial communities causing a shift in gut- and microbe-derived metabolite production (2, 5–9). These consequences may contribute to neoplastic transformations and oncogenesis in a variety of malignancies (10). In a murine model of multiple myeloma, Calcinotto and colleagues (11) found the pathogenic species *Prevotella heparinolytica* was associated with enhanced Th17 cell activation within Peyer patches along the gastrointestinal (GI) tract. These Th17 cells migrated to the bone marrow microenvironment and produced IL-17 which synergized with eosinophils, driving multiple myeloma tumor progression (11). In lymphoma, high-fiber diets increased the short-chain fatty acid butyrate, which was shown to inhibit tumor growth (12). Additionally, the metabolite L-glutamine produced by nitrogen-recycling bacteria, namely *Klebsiella* and *Streptococcus*, was highly enriched in patients with multiple myeloma (13). These recent studies highlight the intertwined relationship between the gut microbiome and certain hematologic malignancies, indicating that a more complete understanding of the gut microbiome in other diseases, including chronic lymphocytic leukemia (CLL), is worthy of investigation.

Chronic antigenic stimulation is believed to drive leukemogenesis in mature lymphoproliferative B cell malignancies, such as CLL (14–16). CLL is a clonally derived B cell malignancy characterized by the accumulation of mature CD5^+^ B cells in the blood, bone marrow, and secondary lymphoid tissues (17). The pathogenesis of CLL is closely related to the activation of critical survival pathways such as the B cell receptor (BCR) and toll-like receptor signaling pathways, in which microbial antigens (e.g., bacterial cell membrane components or unmethylated DNA) are known to be key stimulators driving CLL cell proliferation (15, 18–21). Given the antigen-driven nature and immune dysfunction observed in CLL (22–24), Faitová and colleagues (25) studied the relationship of the microbiome to CLL and demonstrated decreased gut microbial diversity characterized by an increase of the *Bacteroidetes* and *Proteobacteria* phyla and the depletion of short-chain fatty acid–producing bacteria (*Lachnospiraceae* and *Ruminococcaceae*) in patients with CLL compared with healthy controls. In a later study by Faitova and colleagues (26), authors similarly investigated the influence of gut microbiome diversity on CLL disease course. Specifically, this study investigated a potential association between the gut microbiome and CLL development using the Eμ-TCL1 adoptive transfer (AT) mouse model of CLL and stool samples of patients with CLL. Murine studies focused on the impact of low- and high-hygiene housing conditions on gut microbial diversity during disease progression, whereas human studies focused on delineating how disease severity corresponded to differences in bacterial diversity (26). The results revealed that a more aggressive form of CLL was present in mice housed in high-hygiene conditions (26). In addition, patients with CLL with decreased microbiome diversity and increased bacterial taxa linked to poor health such as *Flavonifractor*, *Anaerotruncus*, and *Dialister* genera seemed to suffer worse outcomes from CLL (26).

In the present study, we investigated how gut microbiota composition contributes to and correlates with CLL disease progression. In both the transgenic and syngeneic Eμ-TCL1 murine models of CLL, we demonstrate that a unique and dysbiotic gut microbiome forms as CLL disease develops. Using an antibiotic-mediated microflora ablation approach with the syngeneic Eμ-TCL1 model, we found that leukemic mice receiving antibiotics demonstrated dramatic shifts in gut microbiome community structure and a delayed disease onset compared with leukemic mice receiving water, highlighting the gut microbiome’s relationship to CLL pathogenesis. These findings support the reciprocal relationship between the gut microbiome and CLL disease development and provide a foundation for further investigation.

## Materials and Methods

### Microbiome studies in murine models of CLL

All animal experiments were approved by the University of Nebraska Medical Center Institutional Animal Care and Use Committee. All mice were housed in a pathogen-free vivarium and fed an autoclaved standard chow diet and water *ad libitum* (Teklad autoclaved LM-485 diet; Inotiv).

#### Eμ-TCL1 transgenic mouse model

Eμ-TCL1 transgenic mice on a C57BL/6J background (27) and age-matched wild-type C57BL/6J (WT B6) mice were included in the study. Ear punches were utilized to confirm *TCL1* transgene expression. DNA was extracted using Mouse Direct PCR Kit (Bimake) following the manufacturer’s instructions. WT B6 mice were originally purchased from The Jackson Laboratory (JAX:000664). Both male and female mice were included in each cohort. Eμ-TCL1 mice (*n* = 13; five males, eight females) and WT B6 control mice (*n* = 7; two males, five females) were housed separately and monitored over 12 months, during which blood and fecal samples were collected at 4, 7, 10, and 12 months. At the study end (12 months), mice were euthanized via CO_2_ inhalation (gradual flow rate of 30%–70%) and cervical dislocation. Tissues (blood, spleen, and intestines) were collected for further analysis. Processing details are noted in the following sections.

#### Syngeneic CLL model

To model aggressive CLL, we utilized the Eμ-TCL1 AT model of CLL as previously described (28, 29). In brief, spleen-derived lymphocytes (1e^7^) isolated from a heavily moribund Eμ-TCL1 mouse (i.e., evident splenomegaly, >90% CD45^+^/CD19^+^/CD5^+^ cells in both blood and spleen) were adoptively transferred into female, immunocompetent WT B6 mice (JAX:000664; ∼8 weeks of age) via tail vein injection. Following AT, mice receiving Eμ-TCL1 tumors were monitored until moribund disease (7 weeks after engraftment). Fecal samples were collected on the day of engraftment and every 2 weeks until study end. Leukemic disease burden was evaluated via flow cytometry analysis of percent CD45^+^/CD19^+^/CD5^+^ cells in the blood beginning 1 week after engraftment, followed by biweekly assessment. At the study end (7 weeks after engraftment), mice were euthanized.

#### Antibiotic-mediated gut microflora ablation studies

Female, immunocompetent WT B6 mice (JAX:000664; ∼8 weeks of age) were left to acclimate for 7 days prior to randomization into an antibiotic or water control group. One week before AT of Eμ-TCL1 spleen-derived lymphocytes into recipient WT B6 mice, mice were randomized to receive normal drinking water (*n* = 10) or an antibiotic cocktail (*n* = 10) as previously described with minor modifications (10). In brief, for the first 5 days, mice receiving an antibiotic cocktail were gavaged with vancomycin (50 mg/kg), neomycin (100 mg/kg), metronidazole (100 mg/kg), and amphotericin B (0.001 mg/kg) in 200 μL water/g body weight. In parallel, mice in the antibiotic arm received the following antibiotics in drinking water until study end: vancomycin (0.5 mg/mL), neomycin (0.5 mg/mL), metronidazole (1 mg/mL), amphotericin B (0.0005 mg/mL), and ampicillin (1 mg/mL). Antibiotics were obtained from Sigma-Aldrich and freshly prepared every day for oral gavage. Control mice were gavaged with similar volumes of water (for the first 5 days) and received normal drinking water until study end. Following the 5-day oral gavage, CLL was initiated via AT of Eμ-TCL1 spleen-derived lymphocytes (28, 29), and mice were routinely sampled for blood and fecal pellets at time of engraftment and every 2 weeks until study end. To ensure mice were receiving antibiotics throughout the entirety of the study (∼2.5 months) with no negative side effects, mouse weight (Supplementary Fig. S1A) and volume of water (Supplementary Fig. S1B) consumed per cage were measured every 3 days. Additionally, confirmation of antibiotic delivery was assessed by comparing DNA concentrations of fecal pellets from mice receiving antibiotics versus water (Supplementary Fig. S1C–S1E).

#### Sex as a biological variable

**Figure 1:**
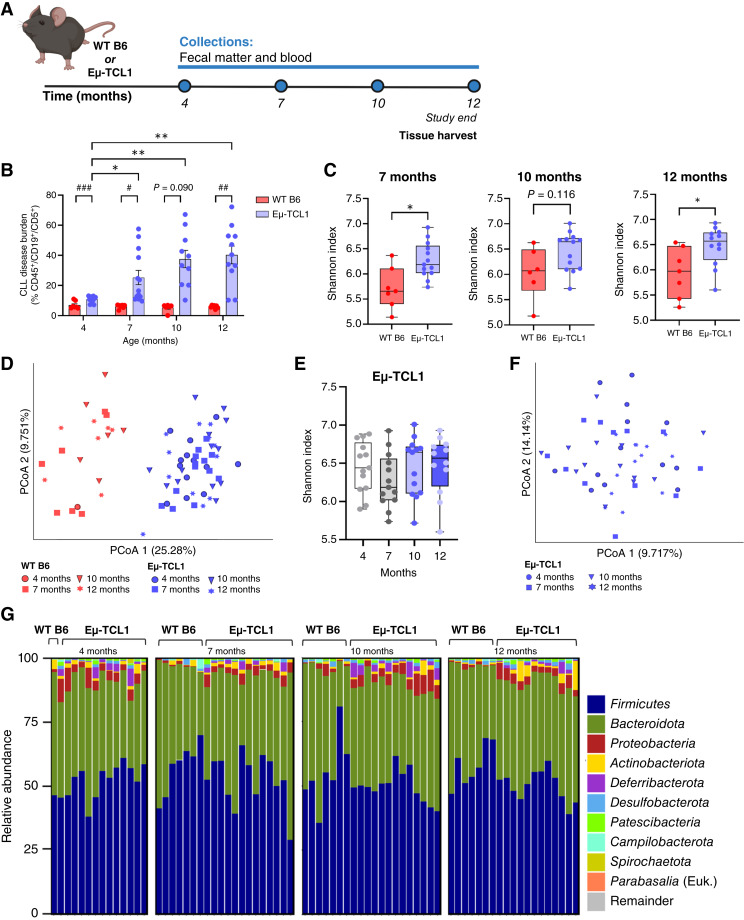
CLL alters gut bacterial diversity and generates a dysbiotic microbiome compared with WT. **A,** Study schematic illustrating transgenic Eμ-TCL1 and WT B6 mice were monitored with serial blood and fecal pellet sample collection over 12 months. DNA isolated from fecal pellets was subject to 16S rRNA sequencing at the indicated time points. At study end, mice were sacrificed, and tissues were harvested (*n* = 7–13 mice/genotype). **B,** CLL disease burden was measured by the percentage of CD45^+^/CD19^+^/CD5^+^ cells in the peripheral blood via flow cytometric analysis. Asterisks denote the significance between Eμ-TCL1 mice over disease course (*, *P* < 0.05; **, *P* < 0.01). Hashtags denote the significance between WT B6 and Eμ-TCL1 mice at each time point (^##^, *P* < 0.01; ^###^, *P* < 0.001). Two-way ANOVA with Dunnett multiple comparisons was applied for testing. **C,** Alpha diversity measured by the Shannon diversity index in transgenic Eμ-TCL1 mice compared with age-matched WT B6 mice at 7, 10, and 12 months (*, *P* < 0.05). Data are presented as box-and-whisker plots, with box edges representing the 25th and 75th percentiles, the center line showing the median value, and whiskers extending to minimum and maximum values. Asterisks denote the significance between WT B6 and Eμ-TCL1 mice at each time point. Unpaired Welch *t* test was applied for testing. **D,** PCoA plot depicting Bray–Curtis distance as a measure of beta diversity between transgenic Eμ-TCL1 mice (blue) and age-matched WT B6 mice (red) at all time points (4 – 12 months). **E,** Alpha diversity measured by the Shannon diversity index in transgenic Eμ-TCL1 mice over time (4–12 months). Data are presented as box-and-whisker plots, with box edges representing the 25th and 75th percentiles, the center line showing the median value, and whiskers extending to minimum and maximum values. **F,** PCoA plot depicting Bray–Curtis distance as a measure of beta diversity in transgenic Eμ-TCL1 mice over time (4–12 months). **G,** Relative abundance bar plots of bacterial taxa present in the gut microbiome of Eμ-TCL1 and WT B6 mice over 12 months. Sequences with a quality score of Q28 and higher were assembled for paired-end sequencing using default parameters of Phredd33 encoding and included in the analysis. Per these parameters, a single 4-month WT B6 sample met this criterion and was retained during this step in the analysis. Additional WT B6 samples in the three subsequent time points (7, 10, and 12 months) passed this quality control check and as such were included. Remainder includes all remaining taxa present in the microbiome at decreased abundance.

**Figure 2:**
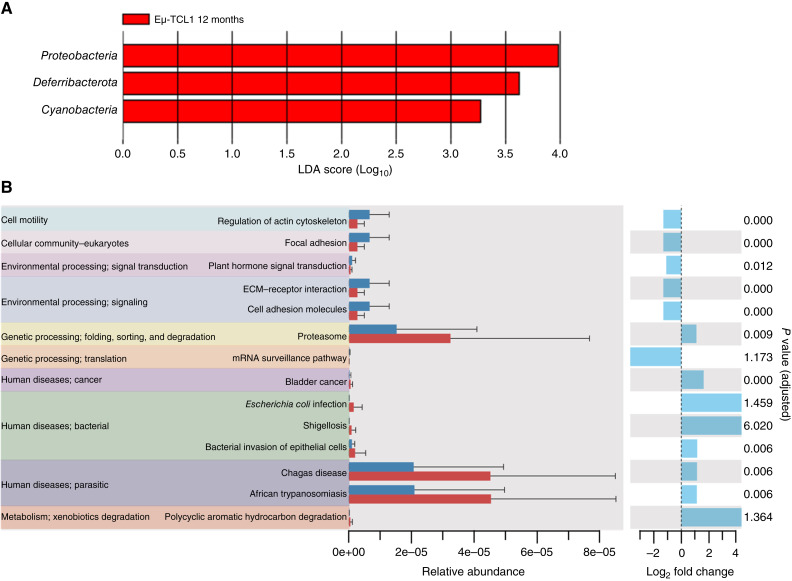
Compositional analysis of microbial communities as CLL disease progresses. **A,** LEfSe analysis demonstrating enrichment of specific bacterial taxa at the phylum level in transgenic Eμ-TCL1 mice (*n* = 12) and WT B6 mice (*n* = 10) at 12 months of age. LDA, linear discriminant analysis. **B,** Predicted microbial function pathways at the phylum level in transgenic Eμ-TCL1 mice and WT B6 mice at 12 months based on 16S rRNA sequencing data analyzed using ggPICRUSt2. ECM, extracellular matrix.

**Figure 3:**
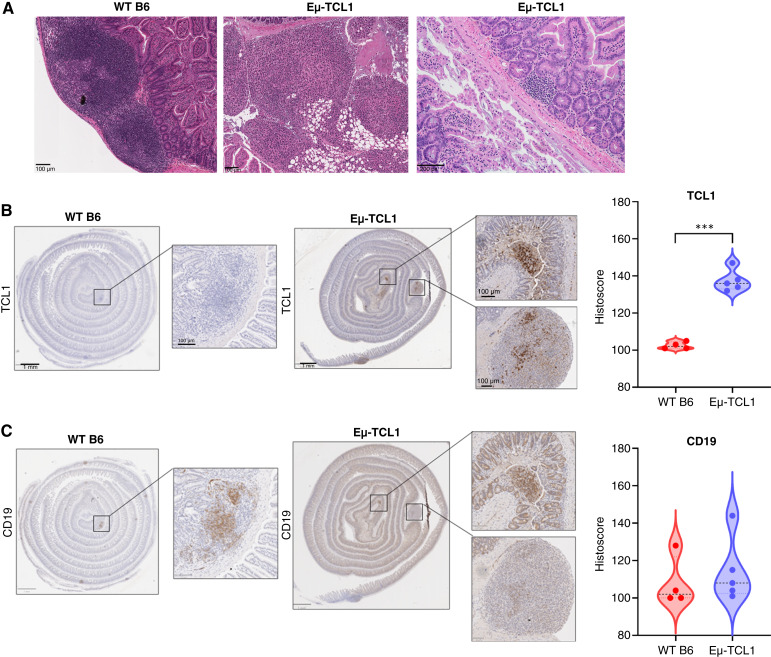
Disturbances in the intestinal tract of Eμ-TCL1 mice with advanced CLL disease. **A,** Representative hematoxylin and eosin images of small intestine tissue in WT B6 and Eμ-TCL1 mice at 12 months (*n* = 7–13 mice/genotype). Hematoxylin and eosin stains: scale bar, 100 μm (left and middle) and 200 pixels (right). **B** and **C,** Representative images of small intestine (serial sections) from 12-month-old WT B6 and Eμ-TCL1 mice (*n* = 4–5 mice/genotype) stained with antibody against human TCL1 (**B**) and murine CD19 (**C**). Histoscore data are presented as violin plots illustrating the empirical distribution of data. The black, dashed line represents the median. Asterisks denote the significance of the histoscores between WT B6 and Eμ-TCL1 mice (***, *P* < 0.001). Unpaired Welch *t* test was applied for testing.

**Figure 4:**
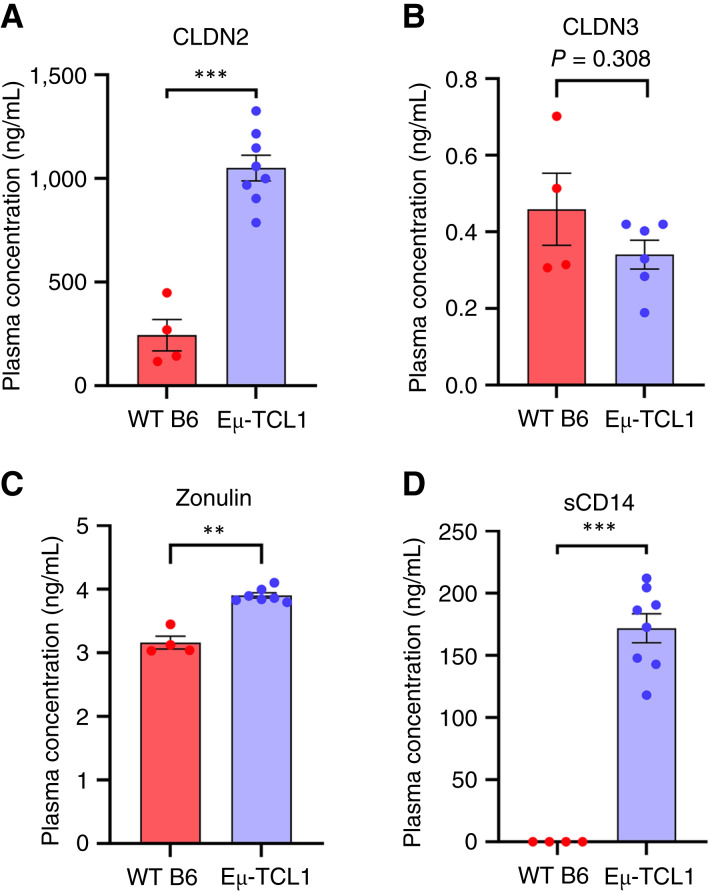
Differential expression of markers pertaining to intestinal integrity and inflammation in WT B6 and Eμ-TCL1 mice. ELISA was used to assess the concentration of tight junction markers in plasma of 12-month-old WT B6 and Eμ-TCL1 mice: CLDN2 (**A**), CLDN3 (**B**), zonulin (**C**), and sCD14 (**D**). Asterisks denote significance between WT B6 and Eμ-TCL1 mice (**, *P* < 0.01; ***, *P* < 0.001). Unpaired Welch *t* test was applied for testing.

**Figure 5:**
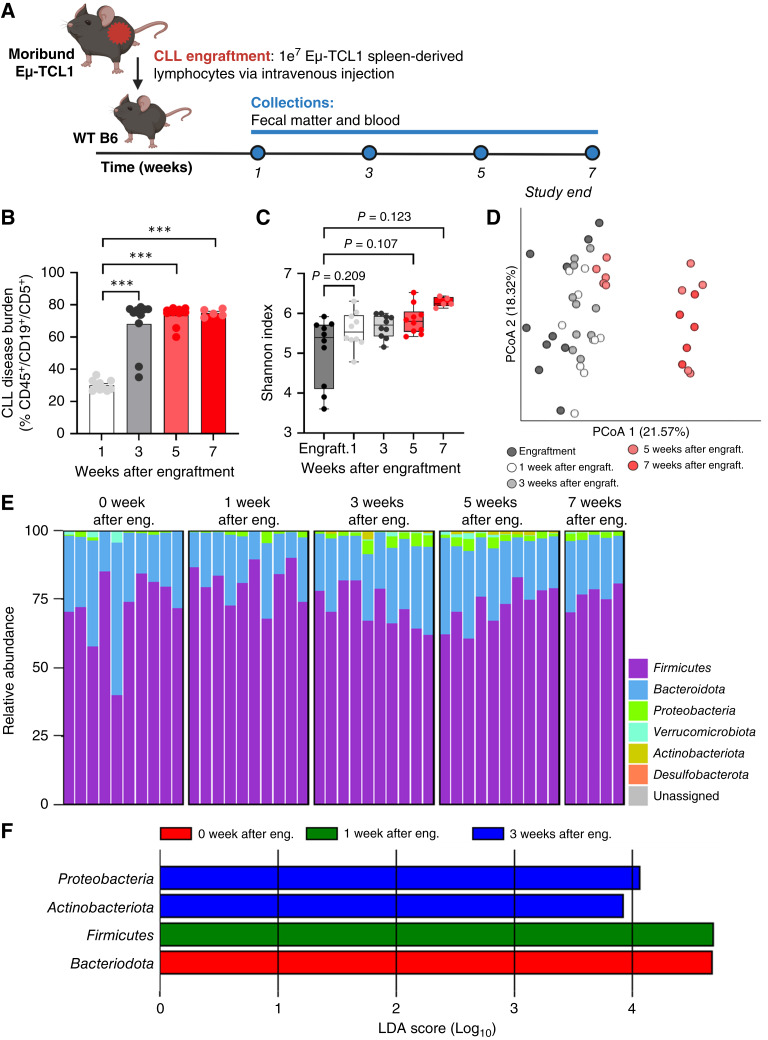
Dysbiotic microbiome is observed in the aggressive syngeneic model of CLL. **A,** Study schematic illustrating WT B6 mice were engrafted intravenously via the tail vein with 1e^7^ spleen-derived lymphocytes from a moribund Eμ-TCL1 mouse (CLL engraftment). Engrafted mice (*n* = 10) were monitored with serial blood and fecal pellet sample collections over the study course of 7 weeks. **B,** CLL disease burden was measured by the percentage of CD45^+^/CD19^+^/CD5^+^ cells in the peripheral blood via flow cytometric analysis starting 1 week after engraftment. Asterisks denote the significance between leukemic mice over time (***, *P* < 0.001). One-way ANOVA with Dunnett multiple comparisons was applied for testing. **C,** Alpha diversity measured by the Shannon index in leukemic mice over 7 weeks. Data are presented as box-and-whisker plots, with box edges representing the 25th and 75th percentiles, the center line showing the median value, and whiskers extending to minimum and maximum values. One-way ANOVA with Dunnett multiple comparisons was applied for testing. **D,** PCoA plot depicting Bray–Curis distance as a measure of beta diversity in leukemic mice over 7 weeks. **E,** Relative abundance bar plots of bacterial taxa present in the gut microbiome of diseased mice over 7 weeks following engraftment. Unassigned includes all remaining taxa present in the microbiome at decreased abundance. **F,** LEfSe analysis demonstrating enrichment of specific bacterial taxa at the phylum level in leukemic mice at the time of engraftment and 1 and 3 weeks after engraftment. Eng./engraft., engraftment. LDA, linear discriminant analysis.

**Figure 6:**
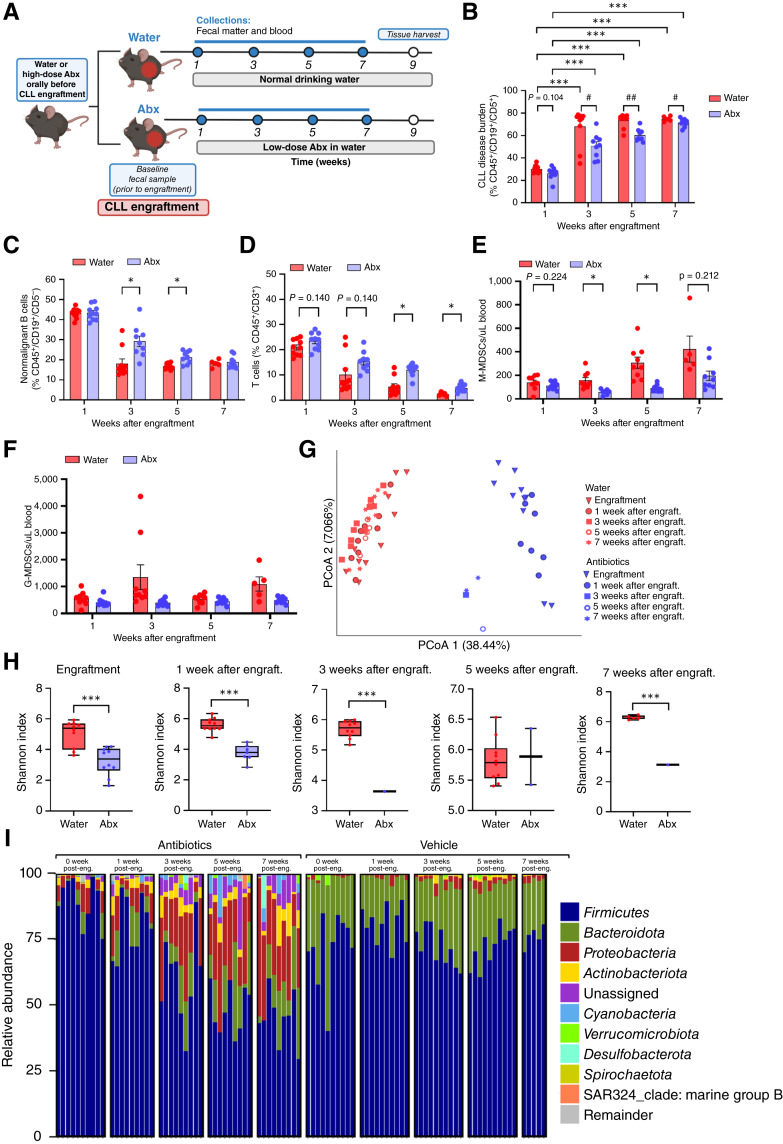
Antibiotic-mediated microflora ablation delays CLL development, fostering a similar dysbiotic microbiome. **A,** WT B6 mice were randomly assigned to either receive an antibiotic cocktail (Abx) or normal drinking water (water). Five days before CLL engraftment, mice receiving antibiotics were gavaged with a high-dose Abx. In parallel, mice in the antibiotic arm received antibiotics in drinking water until study end (left). Control mice were gavaged with similar volumes of water and received normal drinking water until study end (left). Following the initial 5-day oral gavage, a baseline fecal pellet sample was obtained, and CLL was initiated via adoptive transfer of Eμ-TCL1 spleen-derived lymphocytes (right; CLL engraftment). Study arm mice were housed in separate cages and monitored over 7 weeks with serial sampling of blood to monitor CLL burden and fecal pellet sample collection for microbiome profiling (*n* = 10 mice/cohort). Spleens were harvest at 9 weeks after engraftment for further immunophenotyping. **B,** CLL disease burden was measured by the percentage of CD45^+^/CD19^+^/CD5^+^ cells in the peripheral blood via flow cytometric analysis beginning at 1 week after engraftment (*n* = 10 mice/cohort). Asterisks denote the significance between antibiotic-receiving or water-receiving mice over time (*, *P* < 0.05). Hashtags denote the significance between antibiotic-receiving and water-receiving mice at each time point (#, *P* < 0.05; ##, *P* < 0.01). Two-way ANOVA with Dunnett multiple comparisons was applied for testing. **C–F,** Abundances of cell types found in the blood of antibiotic-receiving and water-receiving mice: non-malignant B cells (CD45^+^/CD19^+^/CD5^−^; **C**) T cells (CD45^+^/CD3^+^; **D**) monocytic-like MDSCs (M-MDSCs; CD45^+^/B220^−^/CD3^−^/CD11b^+^/Ly6C^+^/Ly6G^−^; **E**), and granulocytic-like MDSCs (G-MDSCs; CD45^+^/B220−/CD3^−^/CD11b^+^/Ly6C^lo^/Ly6G^+^; **F**). Asterisks denote the significance between antibiotic-receiving and water-receiving mice over time (*, *P* < 0.05). Unpaired Welch *t* test was applied for testing. **G,** PCoA plot depicting Bray–Curtis distance as a measure of beta diversity between Abx (blue) and water (red) arms. **H,** Alpha diversity measured by the Shannon index in antibiotic and water cohorts at time of engraftment and 1, 3, 5, and 7 weeks after engraftment. Data are presented as box-and-whisker plots, with box edges representing the 25th and 75th percentiles, the center line showing the median value, and whiskers extending to minimum and maximum values. Asterisks denote the significance between antibiotic-receiving and water-receiving mice at each time point (***, *P* < 0.001). Unpaired Welch *t* test was applied for testing. **I,** Relative abundance bar plots of bacterial taxa present at the phylum level in the gut microbiome of antibiotic-receiving leukemic mice and water-receiving leukemic mice. Remainder includes all remaining taxa present in the microbiome at decreased abundance. Engraft., engraftment; MDSCs, myeloid-derived suppressor cells; post-eng., after engraftment.

**Figure 7:**
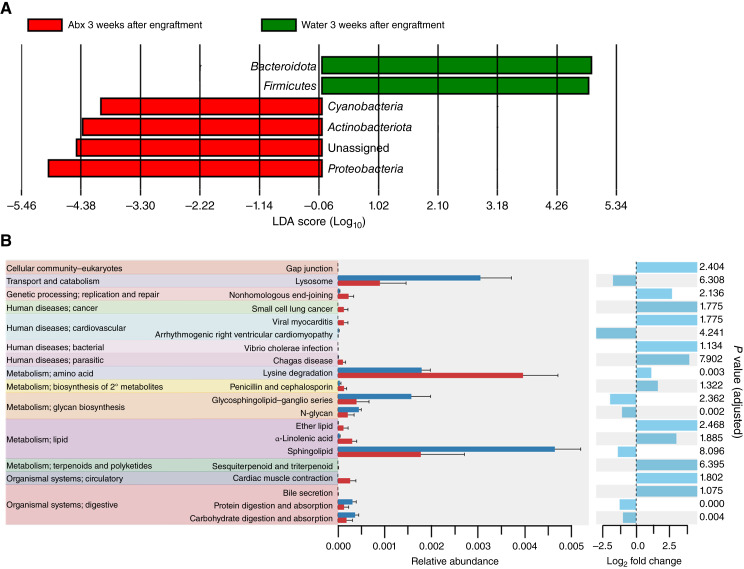
Compositional analysis of microbial communities following antibiotic ablation. **A,** LEfSe analysis demonstrating enrichment of specific bacterial taxa at the phylum level in antibiotic-receiving (Abx) leukemic mice and water-receiving (water) leukemic mice at 3 weeks after engraftment. **B,** Predicted microbial function pathways at the phylum level in antibiotic-receiving leukemic mice and water-receiving mice at 3 weeks after engraftment. LDA, linear discriminant analysis.

CLL is more prevalent in males, with a 1.9:1 male-to-female ratio (30). For the Eμ-TCL1 transgenic study detailed in Figs. 1, 2, 3, and 4 both male and female transgenic Eμ-TCL1 and WT C57BL/6J mice were used. For these experiments, we preferentially used colony mice that were bred in-house following an approved Institutional Animal Care and Use Committee protocol. Therefore, colony logistics and the specific 4-month randomization time point ultimately resulted in more females than males being used in the year-long study. For our Eμ-TCL1 adoptive transfer studies detailed in Figs. 5, 6, and 7, only female mice were used. Because studies have shown that male Eμ-TCL1 mice tend to develop disease later and on average live longer than female mice (31), it is expected that our findings are relevant in male mice.

### Murine sample processing

Murine blood immunophenotyping for routine evaluation of disease status (% CD45^+^/CD19^+^/CD5^+^ cells) was performed using ∼25 μL of blood obtained from the submandibular vein at the previously mentioned time points for each study. At study endpoints, mice were anesthetized with 20% v/v isoflurane (VetOne) in propylene glycol (Thermo Fisher Scientific), and blood was obtained via cardiac puncture. Mice were euthanized via CO_2_ inhalation followed by cervical dislocation to harvest spleens and intestinal tissues. Plasma was isolated from cardiac blood by centrifugation at 2,000 × *g* for 15 minutes at 4°C and stored at −80°C. Spleens, collected into sterile PBS containing 2% heat-inactivated FBS (hi-FBS; Avantor), were homogenized into a single-cell suspension by passing through a 70-μm filter, and then red blood cells were lysed with 1X RBC Lysis Buffer (BioLegend). Freshly isolated murine splenocytes were cryopreserved in 90% hi-FBS containing 10% DMSO. The entirety of the small intestine–cecum–large intestine (colon) was harvested and placed into sterile PBS containing 2% hi-FBS and kept on ice until processing. The cecum was removed from the proximal end of the colon and the distal end of the small intestine, and then the small intestine was cut into three equal sections representing the duodenum, jejunum, and ileum. All three small intestine sections and the colon were cut longitudinally along the mesenteric line and opened with the luminal side facing upward. Remaining fecal matter was carefully removed from the intestinal tissue sections, and each section was washed twice with PBS. Once clean, sections were coated with a layer of 10% neutral buffered formalin and then gently and slowly Swiss-rolled from the distal end to the proximal end. Once rolled, intestinal tissues were placed into a tissue-processing cassette for paraffin embedding and subsequent immunohistochemistry (IHC) or immunofluorescence (IF) staining.

### Tissue histology and immunostaining

IHC and IF staining were performed to evaluate CLL involvement in the GI tract and intestinal barrier integrity, respectively, following standard procedures (32, 33) as detailed below.

Small intestine tissues (5 μm thick) were deparaffinized in Histoclear and rehydrated using a series of graded ethanol solutions (100%, 95%, 70%, and 50%) prior to hematoxylin and eosin staining. For antigen retrieval, slides were incubated in antigen unmasking buffer (Tris-EDTA, pH 9.0) using a pre-set cycle on a medical-grade pressure cooker. Sections were then cooled to room temperature and washed three times with distilled water for 5 minutes. Endogenous peroxidase activity was quenched by incubating the sections in 3% H_2_O_2_ for 10 minutes at room temperature, followed by three additional washes in distilled water (5 minutes each). Sections were incubated with respective antibodies, followed by using VECTASTAIN ABC Kit (Vector Laboratories). Signal development was performed with 3,3′-diaminobenzidine (DAB) chromogen solution (Vector Laboratories). Sections were counterstained using hematoxylin, dehydrated through a graded ethanol series (100%, 95%, and 70%), mounted, and visualized under a light microscope.

For the IHC staining of the small intestine, colon, and tissue-like protrusions, slides with 5-μm-thick tissue sections were deparaffinized in HistoChoice Clearing Agent (cat. #H103-4L; VWR), followed by rehydration using a graded series of ethanol. The slides were subjected to heat-induced antigen retrieval in 10 mmol/L citrate buffer (pH 6.0). After cooling to room temperature, the tissue sections were treated with BLOXALL Blocking Solution (SP-6000) to reduce nonspecific binding sites. Next, the tissue sections were incubated overnight with primary antibody [anti-human TCL1 (CST4042S) 1:400 or anti-mouse CD19 (CST90176) 1:100; Cell Signaling Technologies] prepared in 2.5% normal horse serum. After 24 hours, slides were processed and developed using ImmPRESS Excel Amplified Polymer Kit Peroxidase (anti-rabbit IgG; cat. #MP-7601; Vector Laboratories). For detection and chromogenic reaction, oxidation of the reagent on the slide was observed until the desired brown/red color was reached. Slides were washed in water, counterstained with hematoxylin, and dehydrated in a reverse-graded series of ethanol and two washes with HistoChoice Clearing Agent. After dehydration, coverslips were mounted onto the slide using EMS Rapid Mounting Medium for microscopy.

#### Quantification of IHC

Histoscore analysis was performed using the QuPath bioimaging analysis software (34). Images were imported into an image project folder within QuPath for analysis. Whole tissue sections were annotated with the region of interest feature, and the positive DAB stain analysis feature was utilized to analyze regions of stained tissue. Optical density sum was used for cell detection, and intensity threshold parameters were optimized for each organ group (small intestine, colon, and tissue-like protrusions) and IHC stain (TCL1 and CD19), respectively, with the cell: DAB optical density mean value as score compartment output. Histoscore was calculated using the number of cells staining negative (0), weak (1+), moderate (2+), and strong (3+) and applied to the following equation: Histoscore = (1 × percentage weak positive cells) + (2 × percentage moderate positive cells) + (3 × percentage strong positive cells).

#### IF staining

Detailed methodology can be found in Supplementary Materials and Methods.

### Fecal microbiome sampling

Mice were individually restrained, and fecal pellets were aseptically collected into sterile 1.5-mL Eppendorf tubes. All fecal samples were homogenized using autoclaved Puritan Wooden Applicator/Stirring Sticks (cat. #807; Puritan Medical Products) and immediately placed on dry ice. The number and weight of fecal pellets were documented at the time of collection. All samples were stored at −80°C prior to DNA extraction.

### Fecal DNA extraction

Total bacterial genomic DNA was purified from fecal samples using QIAamp PowerFecal Pro DNA Kit (cat. #51804; QIAGEN) and the TissueLyser LT (cat. #85600; QIAGEN) following the manufacturer’s instructions. The quantity and quality of extracted DNA were assessed using NanoDrop One Microvolume UV-Vis Spectrophotometer (cat. #13400518; Thermo Fisher Scientific). Genomic DNA was stored at −20°C until further analysis.

### Flow cytometry analysis

#### Evaluating circulating tumor burden

For routine monitoring of leukemia progression, ∼25 μL blood was obtained from the submandibular vein. Whole blood was incubated with fluorochrome-labeled antibodies at 4°C for 20 minutes followed by red blood cell lysis with 1X RBC Lysis Buffer (cat. #420301; BioLegend) per the manufacturer’s protocol prior to flow cytometry acquisition. Fluorochrome-labeled antibodies against CD5 (clone 53-7.3; RRID: AB_312735), CD19 (clone 6D5; RRID: AB_313642), and CD45 (clone 30-F11; RRID: AB_312977) were obtained from BioLegend. Flow cytometry acquisition details and flow cytometry gating strategies are found in Supplementary Materials and Methods.

#### Immunophenotyping of peripheral blood

To evaluate additional lymphoid and myeloid immune cell populations, ∼25 μL whole blood was incubated with fluorochrome-labeled antibodies at 4°C for 20 minutes followed by red blood cell lysis with 1× RBC Lysis Buffer (cat. #420301; BioLegend) per the manufacturer’s protocol prior to flow cytometry acquisition. Fluorochrome-labeled antibodies against B220 (clone RA3-6B2; RRID: AB_313004), Ly6C (clone HK1.4; RRID: AB_10897805), Ly6G (clone 1A8; RRID: AB_1186099), CD3 (clone 145-2C11; RRID: AB_312685), CD5 (clone 53-7.3; RRID: AB_312735), CD19 (clone 6D5; RRID: AB_ 313642), and CD45 (clone 30-F11; RRID: AB_312977) were obtained from BioLegend. Flow cytometry acquisition details and flow cytometry gating strategies are found in Supplementary Materials and Methods.

### Bacterial 16S rRNA sequencing and analysis

#### 16S rRNA sequencing

For high-throughput 16S rRNA library preparation and sequencing, the V3-V4 hypervariable region of the 16S gene was amplified from the genomic DNA (12 ng DNA per sample) of murine fecal samples according to the Illumina 16S metagenomics protocol (part #15044223 Rev. B) and sequenced on an Illumina MiSeq platform at the University of Nebraska Medical Center Genomics Core. Detailed methodology is found in Supplementary Materials and Methods.

#### Amplicon data processing

Illumina-generated sequence data were processed using the QIIME2 (v 2022.2) software package (35). Sequences with quality score of Q28 and higher were assembled for paired-end sequencing using default parameters of Phred33 encoding and included in the analysis. Per these parameters, a single 4-month WT B6 sample met this criterion and was retained during this step in the analysis. Additional WT B6 samples in the three later time points (7, 10, and 12 months) passed this quality control check and as such were included. Sequence data were provided to us demultiplexed; therefore, the *demux* command was only run to summarize preliminary descriptive information. Adapter sequences were trimmed using the Cutadapt command, cutadapt v 4.0 (36), and Python v 3.8.13 in QIIME2 (v 2022.2; ref. 35). Filtered sequences were trimmed based on quality and denoised to reduce chimeric sequences using *sklearn* and default DADA2 (37) parameters with parent parameters set to 4. A sampling depth of 8,398 was chosen. Taxonomy was assigned to the identified samples using the SILVA taxonomic classification database (38–40) and visualization output parameter in QIIME2 (v 2022.2; ref. 35).

#### Gut microflora analysis

Relative abundance plots and alpha diversity plots (Shannon index; ref. 41) were generated using the visualization output parameter in QIIME2 (v 2022.2; ref. 35). Relative abundance data were downloaded, and graphs were generated using R (42) and packages *tidyverse* (43) and *qiime2R* (44). Alpha diversity was used as a measure of species richness and evenness between cohorts (transgenic Eμ-TCL1 vs. WT B6; leukemic antibiotic-receiving vs. leukemic water-receiving). Beta diversity was used as a measure of community similarity based on species composition between cohorts. Beta diversity was presented as principal coordinate analysis (PCoA) plots, generated using Bray–Curtis distance metrics (41). Linear discriminant analysis effect size (LEfSe) analysis (45) was used to identify differentially enriched bacterial taxa between cohorts. PICRUSt2 software was used to predict functional abundances of bacterial genes present in microbial community metagenomes (46). The ggpicrust2 package was applied to analyze, interpret, and visualize PICRUSt2 results (47).

### Measurement of plasma markers of tight junction permeability

Plasma levels of claudin-2 (CLDN2; MBS3806619), claudin-3 (CLDN3; MBS764676), soluble CD14 (sCD14; MBS3807351), and zonulin (MBS9363444) were measured using ELISA kits from MyBioSource per the manufacturer’s protocols.

### Statistical analysis

Statistical analyses were performed using GraphPad Prism v 10.4.0 (GraphPad Software). Data are reported as the mean ± SEM unless otherwise indicated. The statistical significance between two groups was determined using unpaired *t* tests coupled to *post hoc* analysis with the Welch correction. The statistical significance between more than two groups was determined using one-way ANOVA or two-way ANOVA coupled with Dunnett multiple comparisons *post hoc* analysis. *P* values less than 0.05 were considered statistically significant.

### Data availability

The sequencing data generated in this study are publicly available in the Sequence Read Archive at the NCBI using BioProject ID PRJNA1187802 at https://www.ncbi.nlm.nih.gov/bioproject/1178702. The code used for the analysis of microbiome data is available at https://github.com/christopherdangelo/DIG-CLL. All other data generated in this study are available upon request from the corresponding author.

## Results

### CLL alters gut bacterial diversity and generates a dysbiotic microbiome

To characterize CLL-associated gut microbiome composition, we first utilized the indolent Eμ-TCL1 mouse model of CLL (27) that reliably captures characteristics of unmutated *IGHV* CLL disease and closely recapitulates the chronic disease progression and immunodeficiencies observed in human CLL. Notably, unmutated CLL BCRs are much more broadly bacteria-reactive than mutated CLL BCRs, making this a suitable model for elucidating the role of the gut microbiota in CLL (48). Eμ-TCL1 mice and age-matched WT B6 mice were monitored over 12 months, which is the expected timeline for Eμ-TCL1 mice to develop moribund disease (Fig. 1A). Expansion of CLL, observed beginning at 4 months of age, was measured by the percentage of CD45^+^/CD19^+^/CD5^+^ cells in the blood (Fig. 1B). Disease burden increased in the Eμ-TCL1 mice at each subsequent time point, with a mean of 36.96% circulating leukemia cells at 12 months (Fig. 1B). To determine the gut microflora diversity in Eμ-TCL1 mice and WT B6 mice, DNA isolated from fecal pellets was analyzed by 16S rRNA sequencing of the V3 to V4 region. Alpha diversity analysis, as measured by the Shannon index (41), showed that gut microbiome communities were distinct between the Eμ-TCL1 and WT B6 cohorts, with Eμ-TCL1 mice exhibiting greater alpha diversity (Fig. 1C). Computed PCoA using Bray–Curtis distance metrics (41) further confirmed distinct differences in the gut microbial communities between Eμ-TCL1 and WT B6 cohorts at all time points (Fig. 1D). However, there is no significant increase in diversity between Eμ-TCL1 mice over time as disease progresses (Fig. 1E and F). Evaluation of the gut microbial communities of Eμ-TCL1 mice at both early (4 months) and late (12 months) disease stages suggests a notable but similar dysbiosis occurring through initiation and progression of disease (Fig. 1G; Supplementary Fig. S2).

To better evaluate differences in the composition and potential function of the microbial communities between the Eμ-TCL1 and WT B6 cohorts, LEfSe and PICRUSt2 algorithms were performed (Fig. 2; refs. 45–47). LEfSe analysis showed enrichment of *Proteobacteria*, *Deferribacterota*, and *Cyanobacteria* phyla at 12 months in Eμ-TCL1 mice compared with WT B6 mice (Fig. 2A). Additionally, the PICRUSt2 analysis demonstrated enrichment of genetic information processing (folding, sorting, and degradation in proteasome) and infectious disease biology (bacterial and bacterial invasion of epithelial cells) in the Eμ-TCL1 cohort (Fig. 2B).

### Intestinal barrier is potentially comprised in Eμ-TCL1 mice with evidence of CLL involvement in the GI tract

In addition to bacterial differences observed in the gut microbiome of diseased Eμ-TCL1 mice and control WT B6 mice, gross examination revealed tissue-like protrusions present on the serosa (mesothelium and epithelium) of the small intestine present exclusively in diseased Eμ-TCL1 mice (Supplementary Fig. S3A). To better characterize these findings, sections of the small intestine were Swiss-rolled and fixed for further histologic evaluation. Multiple transgenic animals contained expansile tumor nodules within the mucosa, bowel wall, or infiltrating the associated adipose tissue composed of large, atypical lymphocytes (Fig. 3A). These findings suggest that the tissue-like protrusions observed were due to the potential involvement of CLL in the GI tract. On further histologic evaluation, despite evidence of CLL involvement, there was no evidence of inflammatory damage to the mucosal surface (Fig. 3A).

Although no evidence of overt inflammation was discovered, we wanted to further understand the impact of both potential CLL infiltration into the GI tract and a dysbiotic microbiome on intestinal barrier integrity. To elucidate any potential CLL involvement in the GI tract, we performed IHC analysis on both WT B6 and Eμ-TCL1 small intestine tissue, colon tissue, and tissue-like protrusions, looking specifically at the expression of human TCL1 and murine CD19. Notably, the expression of TCL1 was significantly increased in the small intestine of Eμ-TCL1 mice compared with WT B6 mice (Fig. 3B). Similarly, TCL1 was markedly expressed in the colon sections of Eμ-TCL1 mice (Supplementary Fig. S3B). Serial tissue sections were also stained for CD19, a marker for normal and neoplastic B cells. As expected, both WT B6 and Eμ-TCL1 mice expressed CD19, but there was no significant difference in histoscores in either the small intestine or colon (Fig. 3C; Supplementary Fig. S3C). We also observe that CD19 tends to colocalize with TCL1-positive cells in the small intestine and colon of Eμ-TCL1 mice (Fig. 3C; Supplementary Fig. S3C). Furthermore, we confirmed the expression of TCL1 and CD19 in the tissue-like protrusions that presented exclusively in the Eμ-TCL1 mice (Supplementary Fig. S3D).

Dysbiosis can lead to changes in intestinal tight junction protein expression and distribution, resulting in altered barrier function (49). We performed IF staining for four intestinal tight junction markers, namely CLDN2, CLDN3, CLDN7, and zonula occludens-1 (Supplementary Fig. S4A and S4B). Within the Eμ-TCL1 cohort, we observed a significant decrease in zonula occludens-1 and a significant increase in CLDN2 expression (Supplementary Fig. S4A and S4B), whereas no changes were observed in CLDN7 or CLDN3 expression (Supplementary Fig. S4A and S4B). Increased expression of CLDN2 frequently occurs during inflammation (50, 51) and is predominantly found in leaky epithelia (52). We further measured plasma levels of markers indicative of tight junction permeability and potential microbial translocation (Fig. 4A–D). We observed significantly higher plasma levels of CLDN2, sCD14, and zonulin in Eμ-TCL1 mice compared with WT B6 mice (Fig. 4A, C, and D). Of note, sCD14 is independently secreted by monocytes in the presence of CLL cells and may separately influence levels (53). CLDN3 was slightly decreased in Eμ-TCL1 mice (Fig. 4B). Taken together, these data confirm the presence of CLL B cells accumulating in the GI tract as well as suggest a remodeling of the epithelial barrier resulting in increased intestinal permeability in diseased Eμ-TCL1 mice.

### Dysbiotic gut microbiome is observed in an aggressive model of CLL

To confirm whether a comparable shift in microbial diversity and composition could be observed in CLL with an aggressive disease course, we next utilized the adoptive transfer Eμ-TCL1 mouse model which closely recapitulates an aggressive form of disseminating CLL with rapid onset and fatal disease within weeks (Fig. 5A). CLL disease was detected in leukemic mice beginning 1 week after engraftment, with disease increasing each week (Fig. 5B). Furthermore, community profiles identified the emergence of a distinct gut microbiome signature in leukemic mice as the disease progressed (Fig. 5C and D). Notably, mice at later disease stages (5 and 7 weeks after engraftment) exhibited increasingly distinctive microbial compositions compared with earlier time points (Fig. 5D). Interestingly, we observed a presence and increase of *Proteobacteria* taxa at 3 and 5 weeks after engraftment (Fig. 5E and F), akin to the aged transgenic Eμ-TCL1 mice. Together, these data confirm that a similar dysbiotic gut microbiome is present in separate indolent and aggressive models of CLL, supporting the validity of this observation and further analysis in our CLL models.

### Antibiotic-mediated gut microflora ablation delays CLL development

Having identified shifts in the gut microbiota as CLL disease progresses, we next tested the impact of directed gut microbial manipulations on CLL outcomes. We adopted an antibiotic-mediated gut microflora ablation approach (10) to the syngeneic Eμ-TCL1 model and compared it with a control Eμ-TCL1 cohort not receiving antibiotics (water cohort; Fig. 6A; refs. 28, 29). In comparison with leukemic mice with intact gut microflora, leukemic mice in the antibiotics arm displayed a significant delay in disease expansion, as evidenced by a lower percentage of CD45^+^/CD19^+^/CD5^+^ peripheral blood lymphocytes at 3 and 5 weeks after engraftment (Fig. 6B). Eventually, leukemic mice receiving antibiotics succumbed to CLL burden comparable with that of leukemic mice receiving water 7 weeks after engraftment (Fig. 6B). Due to this significant delay in CLL disease onset, we sought to better characterize the immune microenvironment of antibiotic- versus water-receiving mice. Percentages of non-malignant B cells decreased over time (Fig. 6C) as the percentage of CLL cells increased (Fig. 6B) in the blood of mice from both arms. Notably, antibiotic-receiving mice housed a greater percentage of non-malignant B cells at the 3- and 5-week time points, corresponding with the significant delay in tumor burden. Additionally, the antibiotic-receiving mice displayed a greater proportion of T cells in the peripheral blood at all time points (Fig. 6D). Antibiotic ablation of the gut microflora also reduced myeloid-derived suppressor cell populations in leukemic mice regardless of whether they took on a more monocytic-like (Fig. 6E) or granulocytic-like (Fig. 6F) phenotype.

We next sought to characterize the immune microenvironment within the spleen, given its role in forming an immunosuppressive tumor niche for CLL cells to engage with various cells in the microenvironment, including T cells and myeloid cells (54). Populations of leukemic and non-malignant B cells were comparable in antibiotic- and water-receiving mice (Supplementary Fig. S5A). Correspondingly, antibiotic- and water-receiving mice displayed slight, non–statistically significant differences in splenic CD4^+^ T cells and CD8^+^ T cells (Supplementary Fig. S5A). However, given the crucial role of antitumoral immune responses by CD8^+^ T cells in CLL (55), we focused on characterizing how the phenotype of CD8^+^ T cells changed when antibiotics were used for gut microflora ablation. Antibiotic-receiving mice displayed minimally reduced T cell dysfunction, evoking a non-significant change in the percentage of total CD8^+^ and antigen-experienced (CD44^+^) CD8^+^ T cells co-expressing inhibitory receptors PD-1, TIM-3, and LAG-3 (Supplementary Fig. S5B). To further characterize the extent of dysfunction, CD8^+^ T cells were classified as progenitor-exhausted T cells (PD-1^int^/TIM-3^lo/-^) or terminally exhausted T cells (PD-1^hi^/TIM-3^hi^; refs. 56, 57). No significant change in percentage of progenitor-exhausted T cells was witnessed, but antibiotic-receiving mice housed a slightly lower proportion of terminally exhausted T cells (Supplementary Fig. S5C). Additionally, total and antigen-experienced CD8^+^ T cells from antibiotic-receiving mice may be more tumor-reactive, as evidenced by a slight elevation in CD39 expression (Supplementary Fig. S5D; refs. 58, 59). Beyond T cells, antibiotic ablation of the gut microflora did not significantly affect any of the myeloid populations evaluated, including myeloid-derived suppressor cells, dendritic cells, or macrophages (Supplementary Fig. S6A–S6D). Overall, more definitive conclusions were not able to be made because of the limited sample size analyzed.

Finally, computed PCoA using Bray–Curtis distance metrics (41) confirmed distinct differences in gut microbial communities in the antibiotic-receiving versus water-receiving cohorts at all time points (Fig. 6G). Alpha diversity analysis, measured by the Shannon index (41), identified reduced diversity in the antibiotic-receiving mice, consistent with known antimicrobial effects on the microbiome (Fig. 6H). These findings suggest that signals from an intact gut microflora may contribute to CLL disease development.

Propelled by the observed delay in CLL disease development and differing microflora composition and diversity observed in the antibiotic-receiving leukemic mice, we sought to identify the specific bacterial taxa present in fecal matter of these mice using 16S rRNA sequencing (Fig. 6I; Supplementary Fig. S7). Initially, the antibiotic microflora ablation regimen produced a shift in the relative abundance of the major phyla. Over time, sustained antibiotic pressure produced a dramatic change in the relative abundance of the microbial community compared with earlier time points for antibiotic- and water-receiving mice. Specifically, *Proteobacteria* and *Bacteroidota* phyla progressively increased in the antibiotics arm throughout the study duration, corresponding with the gradual rise in CLL burden (Fig. 5I; Supplementary Fig. S7B and S7C). Moreover, the following phyla were selectively present in leukemic mice receiving antibiotics: *Actinobacteriota*, *Firmicutes*, and Remainder (i.e., remaining taxa present in microbiome at decreased abundance; Supplementary Fig. S7A, S7D, and S7K). Further compositional analysis of the gut microbial communities showed enrichment of *Bacteroidota* and *Firmicutes* in leukemic mice receiving water and enrichment of *Cyanobacteria*, *Actinobacteria*, and *Proteobacteria* in leukemic mice receiving antibiotics (Fig. 7A). Additionally, PICRUSt2 analysis of antibiotic-receiving mice featured a reduction in bacterial populations associated with N-glycan synthesis, protein digestion, and carbohydrate digestion, and an increase in lysine degradation compared with water-receiving mice (Fig. 7B). These data indicate that antibiotic administration produces an immediate change in the microbiome which may remove potentially pathogenic taxa that drive CLL progression. Over time, sustained antibiotic pressure produces a further change in the gut microbiome, which becomes progressively dysbiotic and features potentially inflammatory taxa, including members of the *Proteobacteria* phylum, which may then facilitate CLL progression.

## Discussion

In this study, we provide results implicating the gut microbiome as a potential factor in the initiation and progression of CLL disease. Within the transgenic Eμ-TCL1 model, as CLL disease progresses over 12 months, we demonstrate a distinct and progressive dysbiotic gut microbiome featuring higher alpha and beta diversities compared with WT B6 controls. Findings from our longitudinal microbiome profiling conflict with previous analysis of fecal samples from patients with CLL (25). In that study, patients were shown to have lower alpha diversity compared with control groups when assessed by the Shannon index (25). Although low alpha diversity of a microbial community typically suggests a pathologic dominance of specific taxa, high alpha diversity by itself may not be inherently indicative of a healthy system, especially when captured at a fixed time point. Instead, higher alpha diversity may highlight a diverse community evolving in conjunction with changes to host physiology (10, 60, 61). This discrepancy could also be due to different experimental models (mice vs. human subjects) or the limited CLL representation in that study (*n* = 10 patients with CLL, *n* = 124 controls; ref. 25). Although controls were matched on criteria such as mean age, residency, year of sampling, and method and frequency of sampling, critical factors such as diet and gender were not accounted for, leaving the possibility of residual confounding.

Factors affecting gut microbial dysbiosis, such as antibiotic administration, are being increasingly investigated for their role in oncogenesis and response to therapy. We found that antibiotic-mediated ablation of the gut microflora facilitated the delayed onset of CLL disease in our study mice, potentially through reducing microbial stimulation of B cells by influencing the microbial load present in the gut microenvironment of leukemic mice receiving the antibiotic cocktail. Immunophenotyping of the splenic tumor microenvironment also suggested that antibiotic-mediated microflora ablation in leukemic mice creates an immune landscape that fosters more tumor-reactive and less exhausted CD8^+^ T cells. Interestingly, antibiotic-treated mice exhibited a significant temporal shift in their gut microbial communities. *Proteobacteria* and *Actinobacteriota* significantly rose in abundance in leukemic mice receiving antibiotics at later study time points (5 and 7 weeks after engraftment). Given the coinciding finding of increased CLL expansion in the blood, these data suggest that these bacteria may serve as markers or influencers of advanced CLL progression. Indeed, an increased abundance of *Proteobacteria* members was separately found across all our experimental murine CLL models, raising speculation that this phylum may contain members that are critical to CLL pathogenesis. *Proteobacteria*, a taxa containing numerous pathogenic members, including the *Pseudomonas* species, are known to be evident in diseases characterized by inflammation, suggesting the presence of this bacteria to be detrimental (62). *Proteobacteria* phyla have also been independently associated with CLL in other microbiome studies (63). In contrast, certain classes of *Actinobacteriota* (i.e., *Bifidobacterium*) have demonstrated beneficial effects in intestinal disease and disorders (64). The relevance of these bacteria in B cell malignancies is not well described; therefore, our results necessitate further investigation into the role of *Proteobacteria* and *Actinobacteria* in CLL, in which redemonstration and additional analysis using qPCR methods can help detect not only relative abundance changes but also absolute changes of these specific taxa within the community.

Although numerous studies have identified associations between gut microbiome features and disease pathogenesis or mitigation, the precise mechanism connecting the microbiome to distant oncologic sites remains unclear. In this study, we identified CLL penetration into GI tissue, evidence of intestinal permeability changes, and alterations in T cell subpopulations. Although these remain as associations at this time, such evidence supports a theory that chronic involvement of CLL cells in the GI tract could be a contributing factor to the development of progressive microbial dysbiosis, which may in turn yield intestinal barrier disruption and bacterial translocation driving antigen exposure and immune cell activation. Support for this mechanism exists from extensive research in human immunodeficiency virus and inflammatory bowel disease, in which human immunodeficiency virus–related enteropathy or inflammatory bowel disease leads to gut epithelial damage, barrier degradation, and subsequent bacterial translocation and immune activation (65–67). These data provide a firm foundation and justification for future studies to explore this connection as a possible mechanism between gut microbiome changes and CLL development.

Our study highlights the presence of a unique and dysbiotic gut microbiome that occurs with the initiation and progression of CLL disease. Although the use of 16S rRNA sequencing is limited by its inability to accurately identify bacterial taxa past the genus–species level, we were able to identify key differences at the phyla level. The experiments described herein are associative at best, and further detailed work examining the roles of the microbiota in general and the specific differential taxa identified needs to be performed to examine casual relationships. Shotgun metagenomics sequencing and metabolomics can provide a more in-depth analysis of specific species, the available genetic repertoire, and metabolite production to further describe the gut microbiota–CLL–immune axis. qPCR studies could also be performed as part of future confirmatory studies of specific taxa changes, which is a limitation of this current analysis. Importantly, our data showing leukemic cell infiltration of the intestinal tissue put forward a previously unrecognized CLL tumor microenvironment that harbors immunologic manifestations on resident gut microbiota, resident immune cells, and gut barrier permeability. This could occur through direct interactions or indirect mediators (i.e., metabolites or soluble factors). Future studies focused on exploring the functional metabolic implication of gut dysbiosis on immune responses and antitumor immunity will enlighten the further characterization of a potential gut microbial signature within CLL pathophysiology.

## Supplementary Material

Supplementary Figure S1 Figure S1. Confirmation of continuous antibiotic delivery in the antibiotic-mediated gut microflora ablation model.

Supplementary Figure S2 Figure S2. Relative abundance of microbiota in Eµ-TCL1 mice versus WT B6 mice.

Supplementary Figure S3 Figure S3. Histopathological analysis of the intestinal tract of Eµ-TCL1 mice with advanced CLL disease.

Supplementary Figure S4 Figure S4. Evidence of intestinal barrier disturbances in Eµ-TCL1 mice with advanced disease.

Supplementary Figure S5 Figure S5. Antibiotic-mediated gut microflora ablation alters T-cell function in leukemic mice.

Supplementary Figure S6 Figure S6. Splenic myeloid cell populations in antibiotic-receiving leukemic mice versus water-receiving leukemic mice.

Supplementary Figure S7 Figure S7. Relative abundance of microbiota in antibiotic-receiving leukemic mice vs. water-receiving leukemic mice.

Supplementary Methods Supplementary Materials and Methods
