# Supplementary material for: Gut Microbiome Profiling in Eμ-TCL1 Mice Reveals Intestinal Changes and a Dysbiotic Signature Specific to Chronic Lymphocytic Leukemia
Source: Cancer Res Commun. 2025 Aug 15;5(8):1344–58. doi: 10.1158/2767-9764.CRC-25-0022 (PMC12354945; doi:10.1158/2767-9764.CRC-25-0022)
Supplement: Supplementary Figure S1 — Figure S1. Confirmation of continuous antibiotic delivery in the antibiotic-mediated gut microflora ablation model. [file crc-25-0022_supplementary_figure_s1_suppsf1.pdf]

Supplementary Figure S1

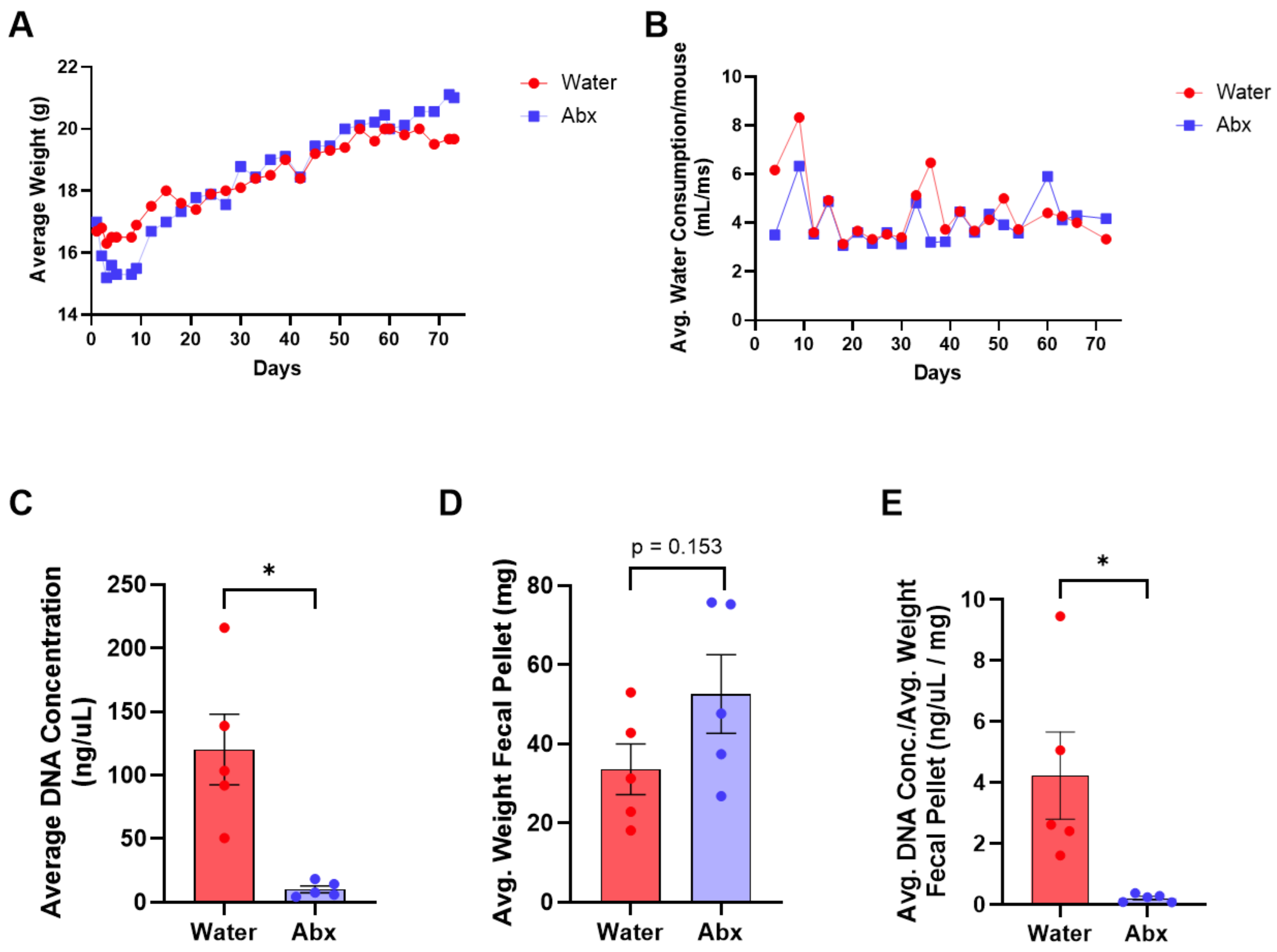

Supplementary Figure S1. Confirmation of continuous antibiotic delivery in the antibiotic-mediated gut microflora ablation model. (A) Average weight (g) of mice. Weight was monitored every three days. **(B)** Average water consumption per mouse. The volume of water (mL) was measured and replenished with fresh antibiotics every three days. Inconsistency with the amount of water drank between antibiotic-receiving and water-receiving mice during the first five days is due to the administration of antibiotics or water via oral gavage. **(C-E)** Evaluation of fecal pellets for average DNA concentration (ng/μL; **C**), average weight of fecal pellets (mg; **D**), and the ratio of average DNA concentration to average weight of fecal pellet (**E**). Asterisks denote the significance between antibiotic-receiving and water-receiving mice over time (* $p < 0.05$). Unpaired Welch's t-test was applied for testing.
