# Supplementary material for: Gut Microbiome Profiling in Eμ-TCL1 Mice Reveals Intestinal Changes and a Dysbiotic Signature Specific to Chronic Lymphocytic Leukemia
Source: Cancer Res Commun. 2025 Aug 15;5(8):1344–58. doi: 10.1158/2767-9764.CRC-25-0022 (PMC12354945; doi:10.1158/2767-9764.CRC-25-0022)
Supplement: Supplementary Figure S2 — Figure S2. Relative abundance of microbiota in Eµ-TCL1 mice versus WT B6 mice. [file crc-25-0022_supplementary_figure_s2_suppsf2.pdf]

Supplementary Figure S2

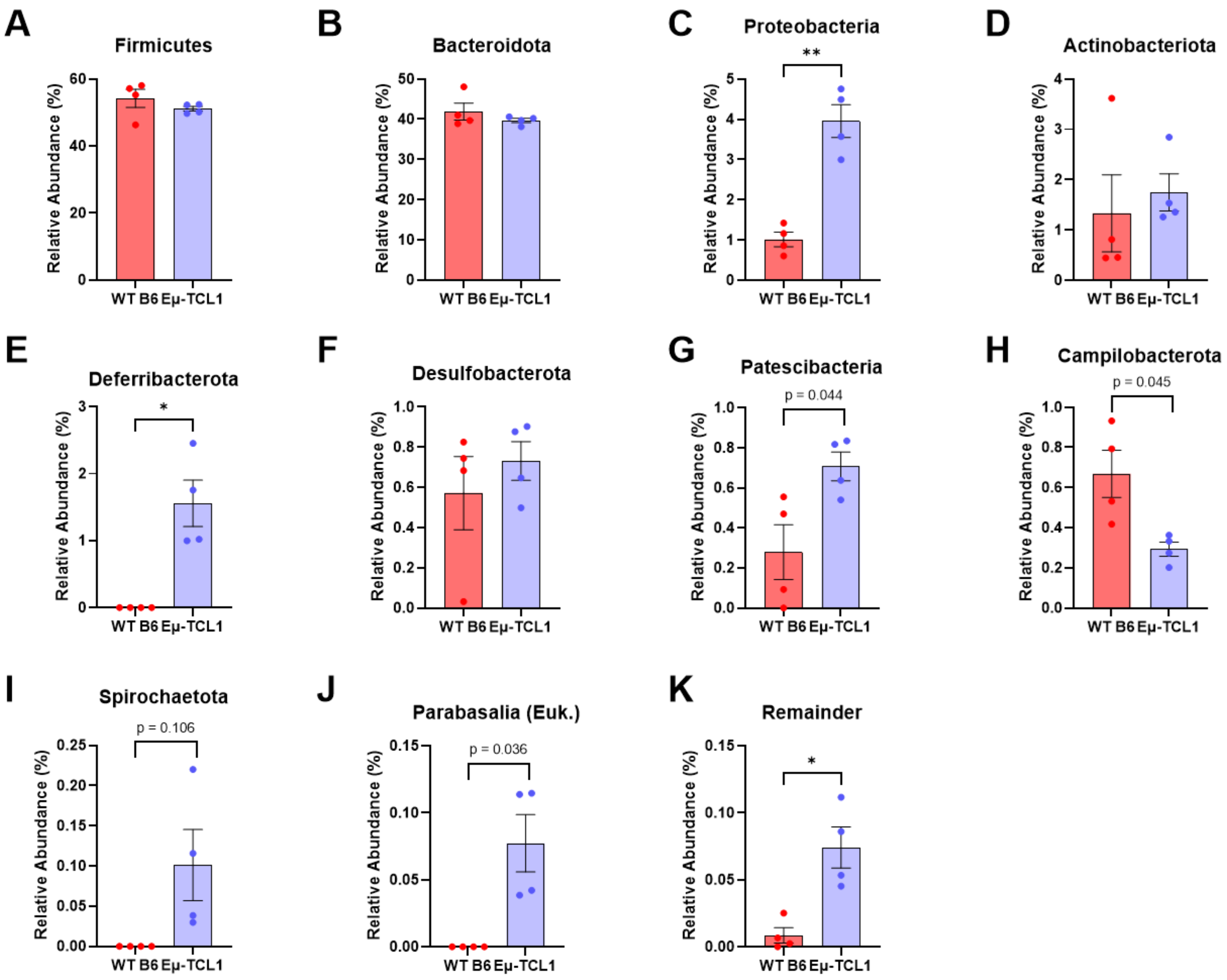

Supplementary Figure S2. Relative abundance of microbiota in Eμ-TCL1 mice versus WT B6 mice. DNA isolated from fecal pellets of transgenic Eμ-TCL1 mice and WT B6 mice from 4, 7, 10, and 12 months of age were subject to 16S rRNA sequencing and compiled. **(A – K)** Relative abundance plots depicting the taxonomic distribution of gut microbiota in transgenic Eμ-TCL1 mice and WT B6 mice (n = 7-13 mice/genotype). Remainder includes all remaining taxa present in the microbiome at decreased abundance. Asterisks denote the significance between Eμ-TCL1 mice WT B6 mice and at all time points (* $p < 0.05$, ** $p < 0.01$). Unpaired Welch's t-test was applied for testing.
