# Supplementary material for: Gut Microbiome Profiling in Eμ-TCL1 Mice Reveals Intestinal Changes and a Dysbiotic Signature Specific to Chronic Lymphocytic Leukemia
Source: Cancer Res Commun. 2025 Aug 15;5(8):1344–58. doi: 10.1158/2767-9764.CRC-25-0022 (PMC12354945; doi:10.1158/2767-9764.CRC-25-0022)
Supplement: Supplementary Figure S3 — Figure S3. Histopathological analysis of the intestinal tract of Eµ-TCL1 mice with advanced CLL disease. [file crc-25-0022_supplementary_figure_s3_suppsf3.pdf]

Supplementary Figure S3

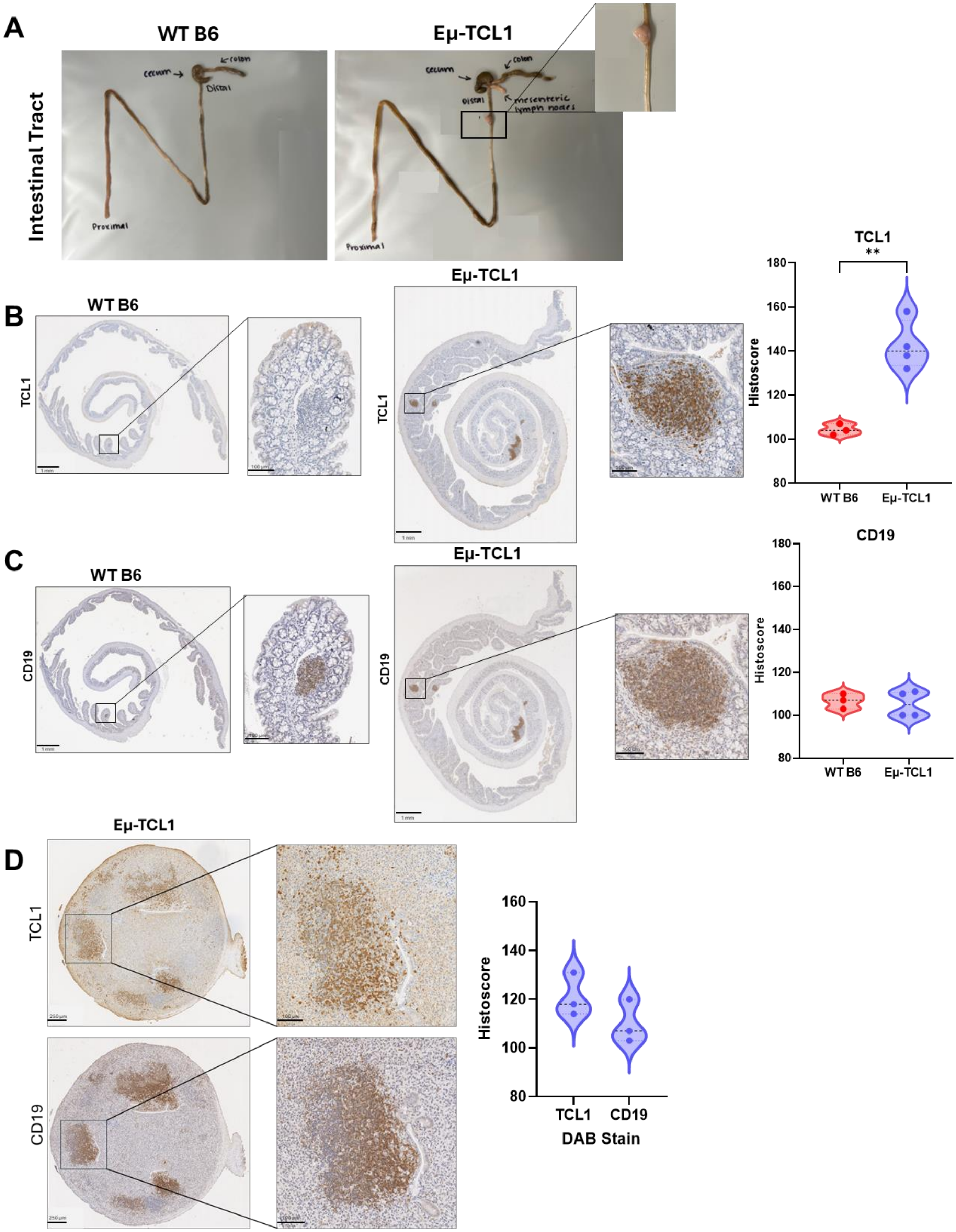

Supplementary Figure S3. Histopathological analysis of the intestinal tract of Eµ-TCL1 mice with advanced CLL disease. (A) Intestinal tracts from Eµ-TCL1 mice with advanced CLL disease (12 months) and age-matched WT B6 mice, harvested, and imaged for gross observation (n = 7-13 mice/genotype). **(B, C)** Representative images (serial sections) of colon from 12-month-old WT B6 and Eµ-TCL1 mice (n = 3-4 mice/genotype) stained with antibody against human TCL1 **(B)** and murine CD19 **(C)**. TCL1 and CD19 stains: magnification 20x, scale bar 1 mm, enlarged area of interest(s) (right), scale bar 100 µm. **(D)** Representative images (serial sections) of tissue-like protrusions from 12-month-old Eµ-TCL1 (n = 3) mice stained with antibody against human TCL1 (top) and murine CD19 (bottom). TCL1 and CD19 stains: magnification 20x, scale bar 250 µm, enlarged areas of interest(s) (right), scale bar 100 µm. Histoscore data are presented as violin plots illustrating the empirical distribution of data. The black, dashed line represents the median. Asterisks denote the significance of the Histoscores between WT B6 and Eµ-TCL1 mice (** $p < 0.01$). Unpaired Welch's t-test was applied for testing.
