# Supplementary material for: Gut Microbiome Profiling in Eμ-TCL1 Mice Reveals Intestinal Changes and a Dysbiotic Signature Specific to Chronic Lymphocytic Leukemia
Source: Cancer Res Commun. 2025 Aug 15;5(8):1344–58. doi: 10.1158/2767-9764.CRC-25-0022 (PMC12354945; doi:10.1158/2767-9764.CRC-25-0022)
Supplement: Supplementary Figure S4 — Figure S4. Evidence of intestinal barrier disturbances in Eµ-TCL1 mice with advanced disease. [file crc-25-0022_supplementary_figure_s4_suppsf4.pdf]

Supplementary Figure S4

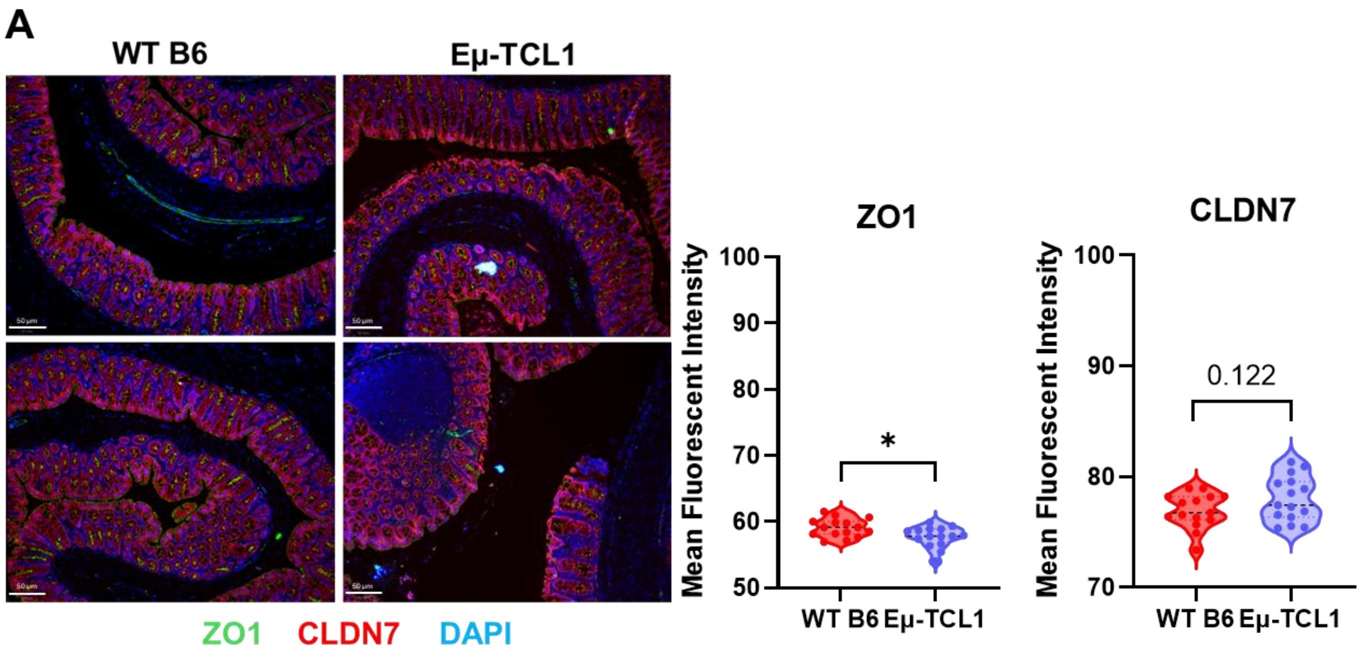

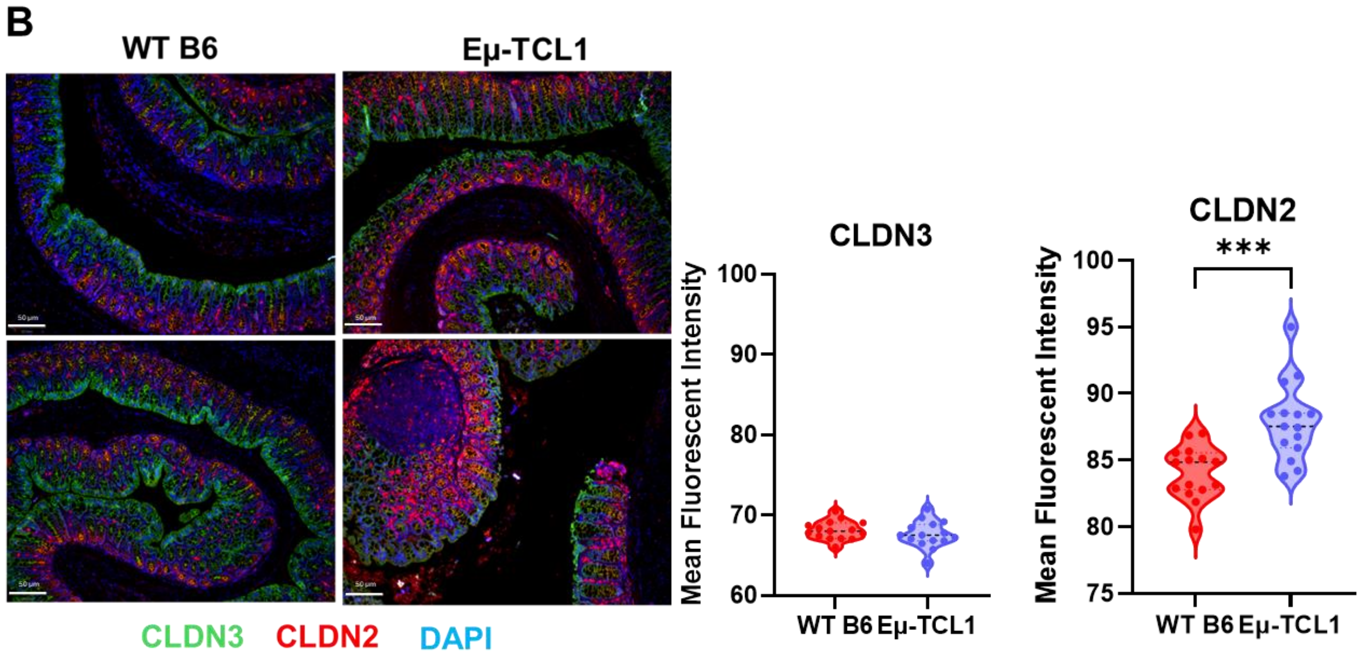

Supplementary Figure S4. Evidence of intestinal barrier disturbances in Eμ-TCL1 mice with advanced disease. (A, B) Representative immunofluorescence images with quantification of colon tissue in WT B6 and Eμ-TCL1 mice at 12 months (n = 3 mice/genotype). Images: magnification 10x, scale bar 50 μm. Tissue sections were stained for zonula occludin-1 (ZO-1, green), claudin-7 (CLDN7, red), and DAPI (blue) **(A)** or claudin-3 (CLDN3, green), claudin-2 (CLDN2, red), and DAPI (blue) **(B)**. Mean fluorescence intensity (MFI) was quantified using FIJI: ImageJ by analyzing five distinct fields per slide. MFI data are presented as violin plots illustrating the empirical distribution of data. The black, dashed line represents the median. Asterisks denote the significance of the MFI values between WT B6 and Eμ-TCL1 mice (* $p < 0.05$, *** $p < 0.001$). Unpaired Welch's t-test was applied for testing.
