# Supplementary material for: Gut Microbiome Profiling in Eμ-TCL1 Mice Reveals Intestinal Changes and a Dysbiotic Signature Specific to Chronic Lymphocytic Leukemia
Source: Cancer Res Commun. 2025 Aug 15;5(8):1344–58. doi: 10.1158/2767-9764.CRC-25-0022 (PMC12354945; doi:10.1158/2767-9764.CRC-25-0022)
Supplement: Supplementary Figure S5 — Figure S5. Antibiotic-mediated gut microflora ablation alters T-cell function in leukemic mice. [file crc-25-0022_supplementary_figure_s5_suppsf5.pdf]

Supplementary Figure S5

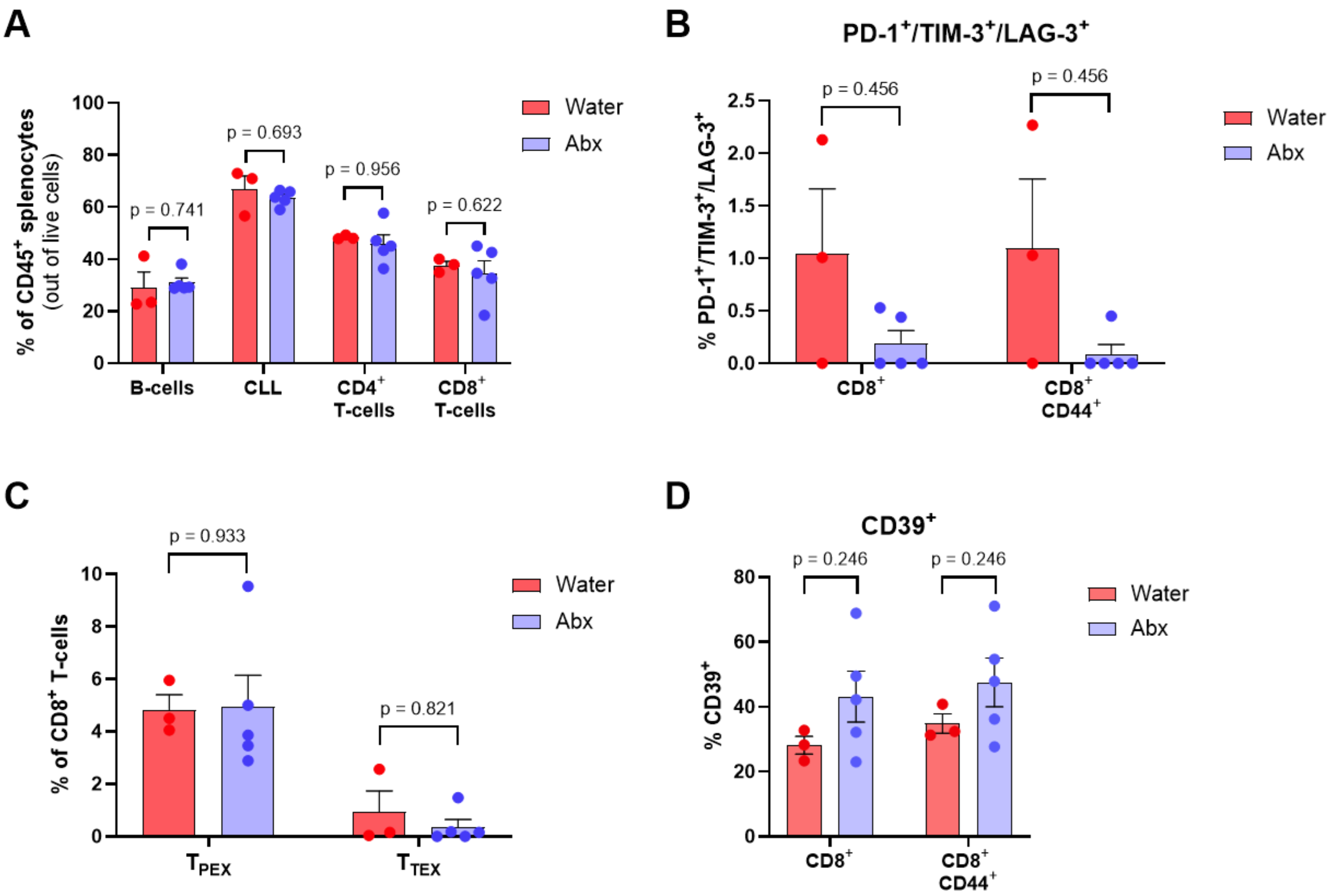

Supplementary Figure S5. Antibiotic-mediated gut microflora ablation alters T-cell function in leukemic mice. (A) Abundances of lymphoid cell types found in the spleen at the time of sacrifice (9 weeks post-engraftment; n = 3-5 mice/cohort). Non-malignant B-cells were gated as CD45$^+$/CD19$^+$/CD5$^-$; CLL cells were gated as CD45$^+$/CD19$^+$/CD5$^+$; T-cells were gated as CD45$^+$/CD19$^-$/CD3$^+$ and either total CD4$^+$ or CD8$^+$ T-cells. Two-way ANOVA with Dunnett's multiple comparisons was applied for testing. **(B)** Percentage of total CD8$^+$ and antigen-experienced (CD44$^+$) CD8$^+$ splenic T-cell subsets co-expressing inhibitory receptors PD-1/TIM-3/LAG-3. Unpaired Welch's t-test was applied for testing. **(C)** Percentage of CD8$^+$ splenic T-cells categorized into progenitor exhausted (T_{PEX}; PD-1^{int}/TIM-3$^{lo/-}$) and terminally exhausted (T_{TEX}; PD-1^{hi}/TIM-3^{hi}). Unpaired Welch's t-test was applied for testing. **(D)** Percentage of total CD8$^+$ and antigen-experienced (CD44$^+$) CD8$^+$ splenic T-cell subsets expressing CD39 as a marker indicative of tumor-reactivity. Unpaired Welch's t-test was applied for testing.
