# Supplementary material for: Gut Microbiome Profiling in Eμ-TCL1 Mice Reveals Intestinal Changes and a Dysbiotic Signature Specific to Chronic Lymphocytic Leukemia
Source: Cancer Res Commun. 2025 Aug 15;5(8):1344–58. doi: 10.1158/2767-9764.CRC-25-0022 (PMC12354945; doi:10.1158/2767-9764.CRC-25-0022)
Supplement: Supplementary Figure S6 — Figure S6. Splenic myeloid cell populations in antibiotic-receiving leukemic mice versus water-receiving leukemic mice. [file crc-25-0022_supplementary_figure_s6_suppsf6.pdf]

Supplementary Figure S6

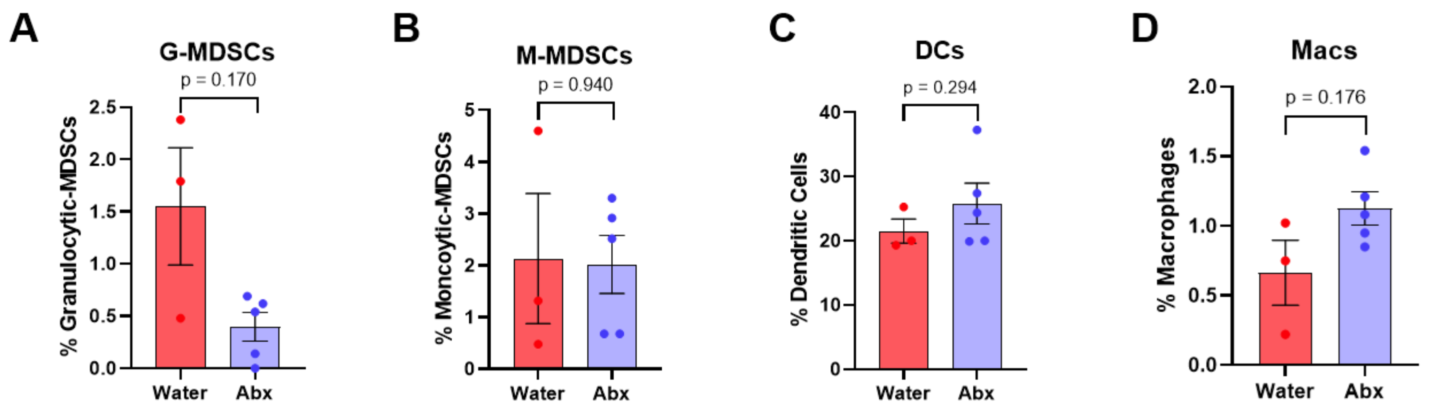

Supplementary Figure S6. Splenic myeloid cell populations in antibiotic-receiving leukemic mice versus water-receiving leukemic mice. (A) Granulocytic MDSCs (G-MDSCs) were gated as $CD19^-/CD3^-/CD11b^+/Ly6C^{lo}/Ly6G^+$ cells in the spleen. **(B)** Monocytic MDSCs (M-MDSCs) were gated as $CD19^-/CD3^-/CD11b^+/Ly6C^+/Ly6G^-$ cells in the spleen. **(C)** Dendritic cells (DCs) were gated as $CD19^-/CD3^-/F4/80^-/MHCII^+/CD11c^+$ in the spleen. **(D)** Macrophages (Macs) were gated as $CD19^-/CD3^-/F4/80^+/CD11b^+$ in the spleen. Unpaired Welch's t-test was applied for testing.
