# Supplementary material for: Gut Microbiome Profiling in Eμ-TCL1 Mice Reveals Intestinal Changes and a Dysbiotic Signature Specific to Chronic Lymphocytic Leukemia
Source: Cancer Res Commun. 2025 Aug 15;5(8):1344–58. doi: 10.1158/2767-9764.CRC-25-0022 (PMC12354945; doi:10.1158/2767-9764.CRC-25-0022)
Supplement: Supplementary Figure S7 — Figure S7. Relative abundance of microbiota in antibiotic-receiving leukemic mice vs. water-receiving leukemic mice. [file crc-25-0022_supplementary_figure_s7_suppsf7.pdf]

Supplementary Figure S7

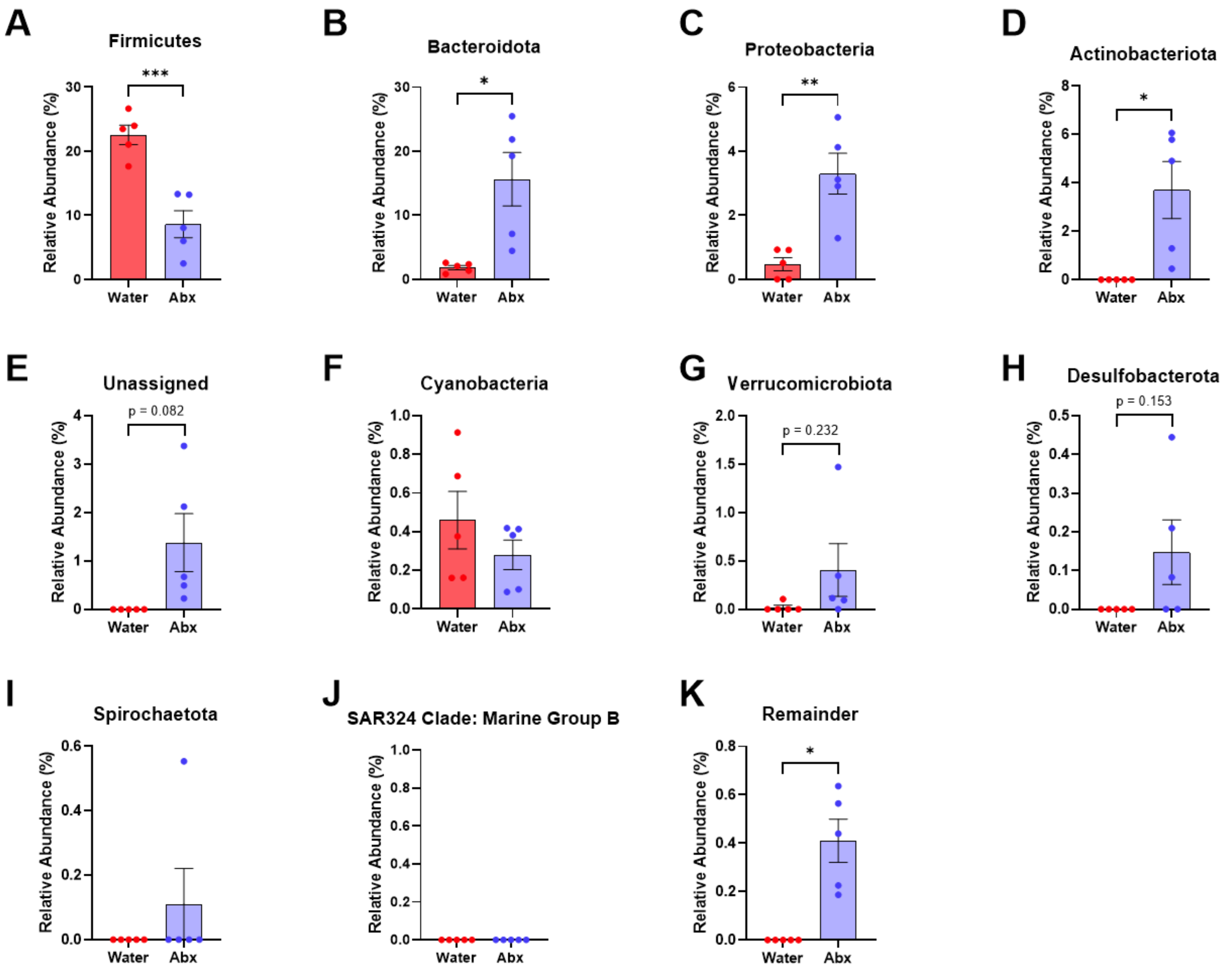

Supplementary Figure S7. Relative abundance of microbiota in antibiotic-receiving leukemic mice *vs.* water-receiving leukemic mice. DNA isolated from fecal pellets of antibiotic-receiving (Abx) leukemic mice and water-receiving (Water) leukemic mice were subject to 16S rRNA sequencing. **(A – K)** Relative abundance plots depicting the taxonomic distribution of gut microbiota in antibiotic-receiving leukemic mice and water-receiving leukemic mice (n = 10 mice/cohort). Remainder includes all remaining taxa present in the microbiome at decreased abundance. Asterisks denote the significance between antibiotic-receiving and water-receiving mice at all time points (* $p < 0.05$, ** $p < 0.01$). Unpaired Welch's t-test was applied for testing.
