# Supplementary material for: Gut Microbiome Profiling in Eμ-TCL1 Mice Reveals Intestinal Changes and a Dysbiotic Signature Specific to Chronic Lymphocytic Leukemia
Source: Cancer Res Commun. 2025 Aug 15;5(8):1344–58. doi: 10.1158/2767-9764.CRC-25-0022 (PMC12354945; doi:10.1158/2767-9764.CRC-25-0022)
Supplement: Supplementary Methods — Supplementary Materials and Methods [file crc-25-0022_supplementary_methods_suppsmm.pdf]

Supplementary Methods

Immunofluorescence analysis

Formalin-fixed, paraffin-embedded colon tissues (5 µm thick) were processed through hydration using routine immunofluorescence protocol. The tissue sections were incubated with respective primary antibodies followed by Cya-3 or FITC-conjugated secondary antibodies (Jackson ImmunoResearch; West Grove, PA, USA). Primary antibodies claudin-2 (Cat. #37-4800), claudin-3 (Cat. #34-1700), and claudin-7 (Cat. #32-5600) were obtained from ThermoFisher (Waltham, MA, USA) and ZO-1 (Cat. #21773-1-AP) was obtained from Proteintech (Rosemont, IL, USA). Nuclei were visualized with DAPI staining (Vector Laboratories; Burlingame, CA, USA).

Immunofluorescence mean intensity quantification was performed using FIGI: ImageJ. TIFF files with multi-fluorophore labeled antibodies were imported into FIGI and color channels were split into independent channels. As intensity threshold was set for each independent color channel. The mean fluorescence intensity of the whole tissue area was measured by using the "Measure" tool within the FIJI platform. Five distinct fields per slide were quantified for each sample. Values were summarized and reported in a violin plot to be evaluated for statistical significance.

Flow cytometry analysis

Immunophenotyping of splenic immune cell populations: Previously isolated and cryopreserved murine splenocytes (~2-3 e^6) were thawed in PBS/2% hi-FBS and incubated with fluorochrome-labeled antibodies at 4°C for 20 minutes. Fluorochrome-labeled antibodies against LAG-3 (C9B7W; RRID: AB_10639935), PD-1 (clone 29F.1A12; RRID: AB_2561447), TIM-3 (clone 5D2; RRID: AB_2744186), CD4 (clone RM4-5; RRID: AB_893326), CD5 (53-7.3; RRID: AB_312735), CD8 (clone 53-6.7; RRID: AB_2563057), CD19 (clone 6D5; RRID: AB_313642), CD39 (cloneDuha59; RRID: AB_2563393), CD44 (clone IM7; RRID: AB_2562451), CD45 (clone 30-F11; RRID: AB_2564590), and Zombie NIR™ Fixable Viability Kit were obtained from BioLegend (San Diego, CA, USA).

Flow cytometry gating strategies: Flow cytometry was performed on a NovoCyte 2060R cytometer (Agilent; Santa Clara, CA, USA) or an LSRFortessa X-50 (BD Biosciences; San Jose, CA, USA). Single lymphocytes were gated by forward and side scatter. Live cells were gated by Live/Dead Near IR (Invitrogen; Waltham, MA, USA) negative staining. Correct compensation, proper acquisition setup, and positive gates based on appropriate fluorescence minus one (FMO) controls were ensured. Data were analyzed using NovoExpress

v1 3.0 software (Agilent; Santa Clara, CA, USA) or Kaluza v2.1 (Beckman Coulter; Brea, CA, USA). Results are expressed as the proportion of cells expressive antigens of interest (number or percent gated positive). Flow cytometry gating strategies for murine blood flow and murine spleen flow are detailed below:

i – Murine blood flow cytometry gating strategies: lymphoid panel

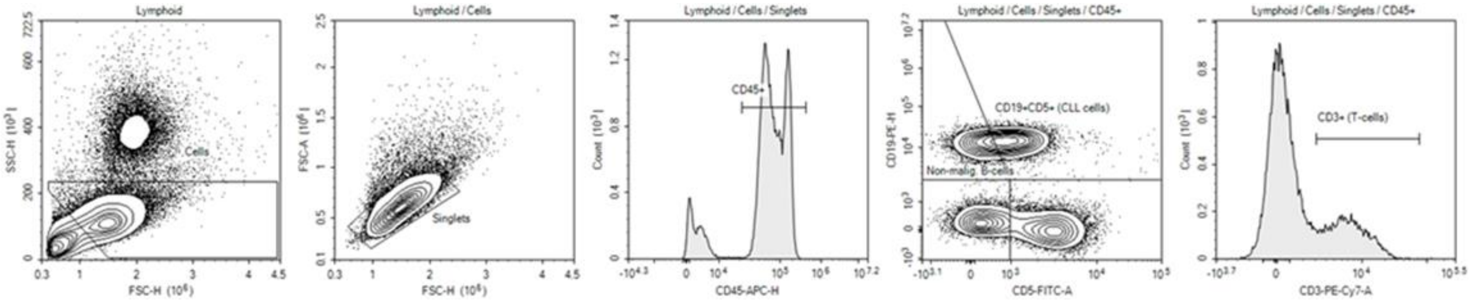

ii – Murine blood flow cytometry gating strategies: myeloid panel

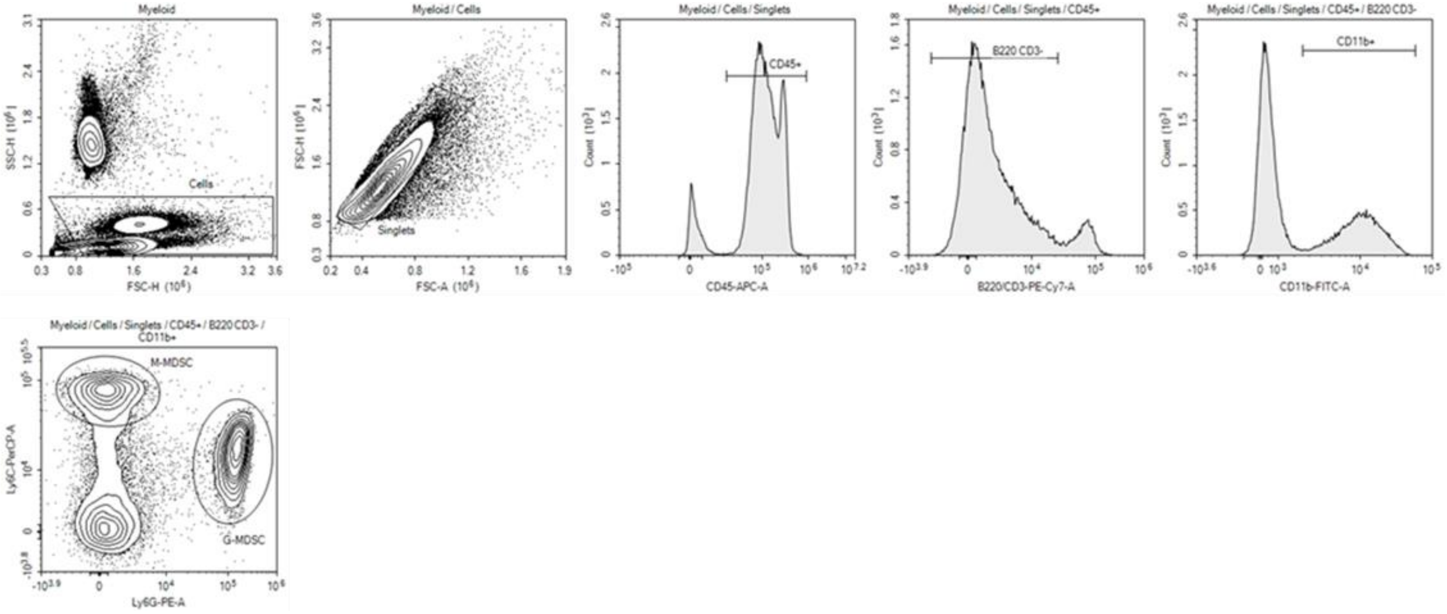

iii – Murine spleen flow cytometry gating strategies: lymphoid panel

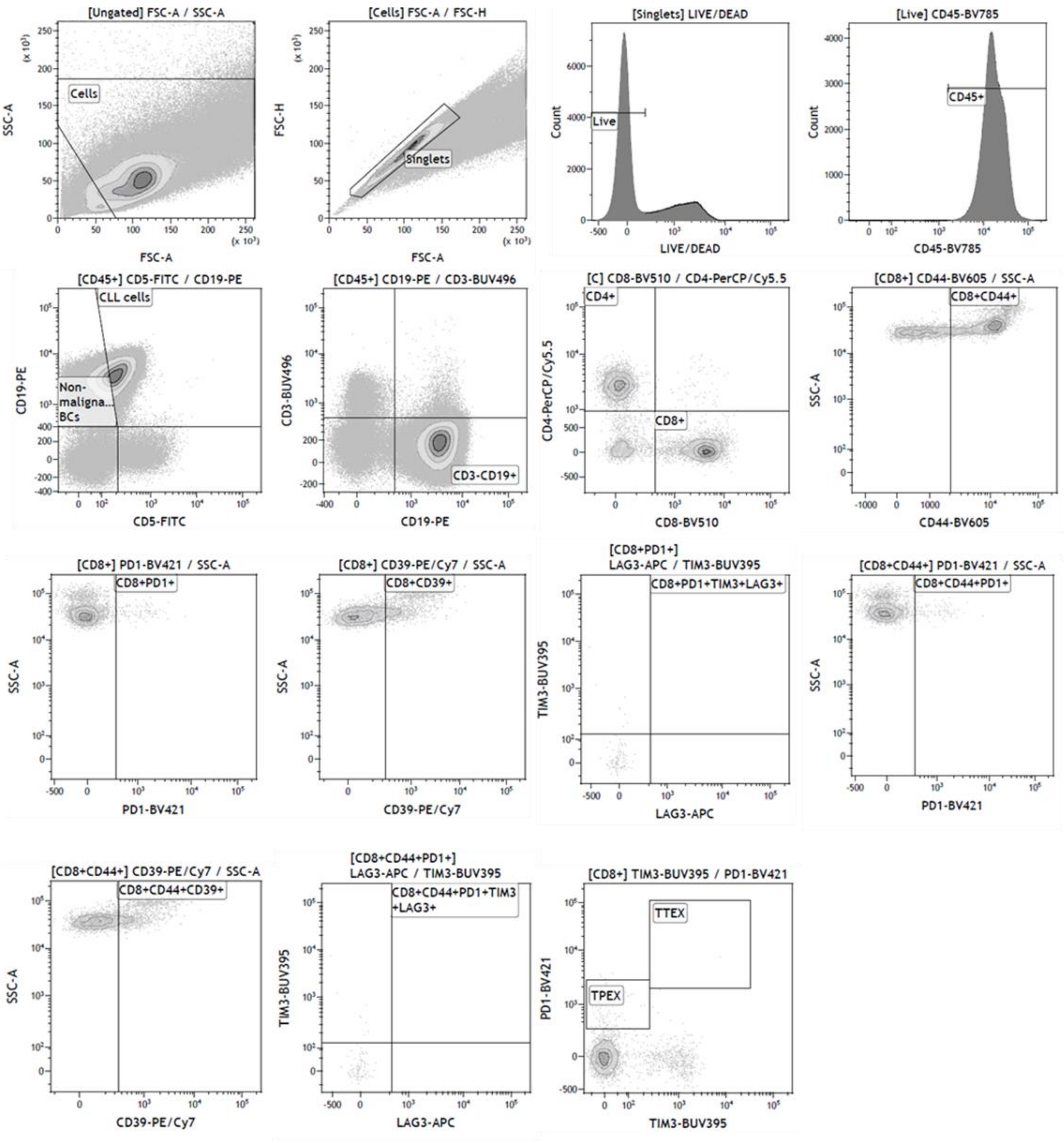

iv – Murine spleen flow cytometry gating strategies: myeloid panel

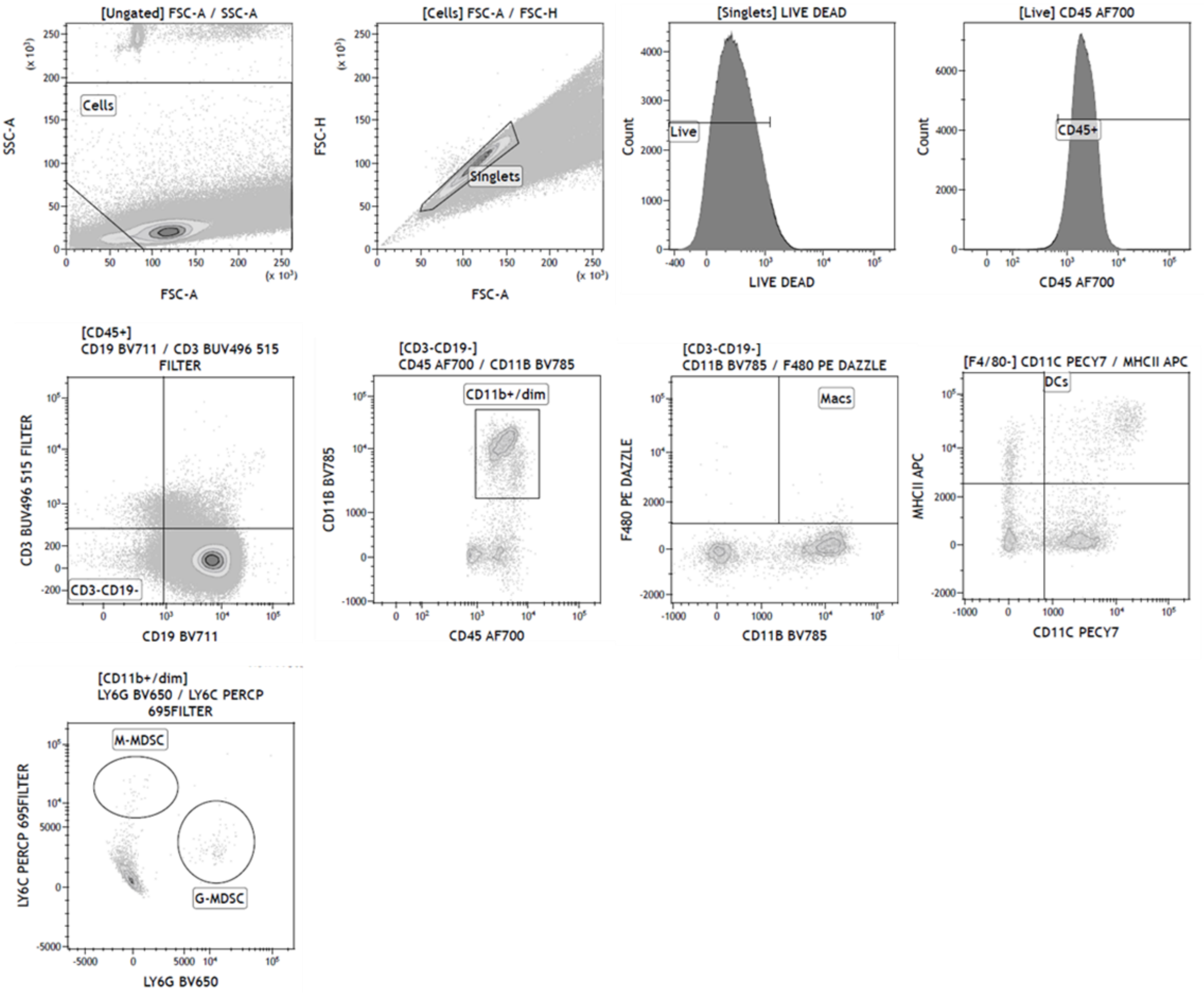

Bacterial 16S rRNA gene sequencing analysis

For high-throughput 16S rRNA library preparation and sequencing, the V3-V4 hypervariable region of the 16S gene was amplified from the genomic DNA (12 ng DNA per sample) of murine fecal samples according to the Illumina 16S metagenomics protocol (Part #15044223 Rev. B) and sequenced on an Illumina MiSeq platform at the UNMC Genomics Core. PCR was performed using the 16S Amplicon PCR primer set (Forward Primer 5’ TCGTCGGCAGCGTCAGATGTGTATAAGAGACAGCCTACGGGNGGCWGCAG and Reverse Primer 5’ GTCTCGTGGGCTCGGAGATGTGTATAAGAGACAGGACTACHVGGGTATCTAATCC) each with adaptor sequences. Samples were amplified (12 ng DNA per sample) in duplicates using 2X KAPA HiFi HotStart Ready

Mix DNA polymerase and purified using AMPure XP beads. Amplification was performed at 95°C (3 minutes) with 25 cycles of 95°C (30 seconds), 55°C (30 seconds), 72°C (30 seconds), and a final extension of 72°C (5 minutes). Dual indices and Illumina sequencing adaptors from Nextera XT Index kits were added to target amplicons in a second index PCR using 2X KAPA HiFi HotStart Ready Mix DNA polymerase. PCR conditions were 95°C (3 minutes), with 8 cycles of 95°C (30 seconds), 55°C (30 seconds), 72°C (30 seconds), and a final extension of 72°C (5 minutes). Final libraries were purified using AMPure XP beads and checked for purity using a Quibit fluorometer. Negative controls were included in all sequencing runs. The pooled amplicon library was denatured with sodium hydroxide (NaOH), diluted with hybridization buffer, heat denatured, and sequenced on an Illumina MiSeq platform using MiSeq Reagent Kit v3 (600 cycles) following the 2 x 300-bp paired-end sequencing protocol.
